# A Lepidopteran-Specific Gene Family Encoding Valine-Rich Midgut Proteins 

**DOI:** 10.1371/journal.pone.0082015

**Published:** 2013-11-29

**Authors:** Jothini Odman-Naresh, Margret Duevel, Subbaratnam Muthukrishnan, Hans Merzendorfer

**Affiliations:** 1 Department of Biology, Chemistry, University of Osnabrück, Osnabrück, Germany; 2 Department of Biochemistry and Molecular Biophysics, Kansas State University, Manhattan, Kansas, United States of America; The Ohio State University/OARDC, United States of America

## Abstract

Many lepidopteran larvae are serious agricultural pests due to their feeding activity. Digestion of the plant diet occurs mainly in the midgut and is facilitated by the peritrophic matrix (PM), an extracellular sac-like structure, which lines the midgut epithelium and creates different digestive compartments. The PM is attracting increasing attention to control lepidopteran pests by interfering with this vital function. To identify novel PM components and thus potential targets for insecticides, we performed an immunoscreening with anti-PM antibodies using an expression library representing the larval midgut transcriptome of the tobacco hornworm, *Manduca sexta*. We identified three cDNAs encoding valine-rich midgut proteins of *M. sexta* (MsVmps), which appear to be loosely associated with the PM. They are members of a lepidopteran-specific family of nine *VMP* genes, which are exclusively expressed in larval stages in *M. sexta*. Most of the *MsVMP* transcripts are detected in the posterior midgut, with the highest levels observed for *MsVMP1*. To obtain further insight into Vmp function, we expressed *MsVMP1* in insect cells and purified the recombinant protein. Lectin staining and glycosidase treatment indicated that MsVmp1 is highly *O*-glycosylated. In line with results from qPCR, immunoblots revealed that MsVmp1 amounts are highest in feeding larvae, while MsVmp1 is undetectable in starving and molting larvae. Finally using immunocytochemistry, we demonstrated that MsVmp1 localizes to the cytosol of columnar cells, which secrete MsVmp1 into the ectoperitrophic space in feeding larvae. In starving and molting larvae, MsVmp1 is found in the gut lumen, suggesting that the PM has increased its permeability. The present study demonstrates that lepidopteran species including many agricultural pests have evolved a set of unique proteins that are not found in any other taxon and thus may reflect an important adaptation in the highly specialized lepidopteran digestive tract facing particular immune challenges.

## Introduction

Many lepidopteran species are serious pests in agriculture and forestry. While adult moths and butterflies are frequently beneficial pollinators that feed on nectar using their siphoning proboscis, caterpillars have chewing mouthparts which allow them to feed on leaf material causing significant plant damage due to defoliation. Rapid growth and development of lepidopteran insects requires the ingestion of large quantities of food to build up sufficient energy reserves stored in the larval fat body, which finally fuel molting and metamorphosis. Caterpillars have a highly efficient digestive system, which has been optimized during evolution to deal with a diet relatively poor in energy yield. In accordance with its vital function, the digestive tract is usually the most prominent organ of the caterpillar, with the midgut being the main digestive compartment. The tobacco hornworm, *Manduca sexta*, is a widely used model organism for studying midgut physiology in lepidopteran insects. The pH of the *Manduca* midgut can reach extremely high values of >11, particularly in the anterior region, due to the combined activity of an electrogenic K^+^/2H^+^ antiporter and a V-type ATPase, both of which reside in the apical membranes of goblet cells [1,2]. The highly alkaline pH is thought to be an adaptation to protect against cross-linking by tannins released from the ingested plant material during maceration [3]. However, it seems likely that lepidopteran insects benefit also from the higher kinetic rates of hydrolytic reactions involving digestive enzymes that have alkaline pH optima such as α-amylase, trypsin or chymotrypsin [4]. Moreover, the high pH may prevent growth of microbial pathogens. On the other hand, it also facilitates activation of toxins such as the σ-endotoxins of *Bacillus thuringiensis* [5], and helps to dissociate viral occlusion bodies to release infectious virions [6]. Digestion of the diet is facilitated by a semipermeable peritrophic matrix (PM), a sac-like structure, which completely envelops the food bolus. In caterpillars, the PM is delaminated by columnar cells of the epithelium along the entire midgut (type I PM) [7]. The PM separates the midgut into endoperitrophic and ectoperitrophic digestive compartments. By partitioning different-sized substrates, and exo- and endo-cleaving digestive enzymes between these two physically separate compartments, the efficiency of hydrolytic reactions is largely increased. Moreover, the PM is thought to protect against mechanical abrasion and enteric pathogens [8]. The PM is made of chitin fibrils, proteins and glycoproteins. Chitin fibrils are synthesized during feeding periods by the midgut-specific chitin synthase 2 (Chs2), which resides in the apical tips of the microvilli forming the brush border of the larval midgut from *M. sexta* [9]. The gene that encodes Chs2 is more highly expressed in the anterior than in the posterior midgut, and regulated during development [10,11]. The proteins that make up the PM are secreted by midgut columnar cells as well. Some of these proteins are referred to as PM proteins (PMPs) [12], and their production varies along the midgut, as shown recently for the larvae of red flour beetle, *Tribolium castaneum* [13]. They have a variable number of chitin-binding domains with six conserved cysteine residues (CBM14 domains), which may be involved in higher order assembly of the chitin fibrils. The PM further contains highly glycosylated proteins with serine-/threonine-rich regions known as invertebrate intestinal mucins (IIMs) [14]. In addition to PMPs and IIMs, there are a greater number of proteins that are more loosely associated with the PM. Some of them may have pivotal functions, and others may be merely trapped within the porous meshwork of the PM [12]. 

To identify novel proteins that are more or less tightly associated with the PM, we performed an unbiased immunoscreening using polyclonal antibodies directed to the purified PM and a λ-Zap cDNA expression library, representing the larval midgut transcriptome of *M. sexta*. In addition to several known classes of midgut proteins, we found a novel class of valine-rich midgut proteins (Vmps), which were originally described as hypothetical cuticle proteins in *Bombyx mori*. We show that these proteins are unique to lepidopteran species and that their distribution within the gut lumen is dramatically altered during starvation and molting.

## Results

### Immunoscreening reveals cDNAs encoding lepidopteran-specific valine-rich midgut proteins (Vmps)

**Figure 1:**
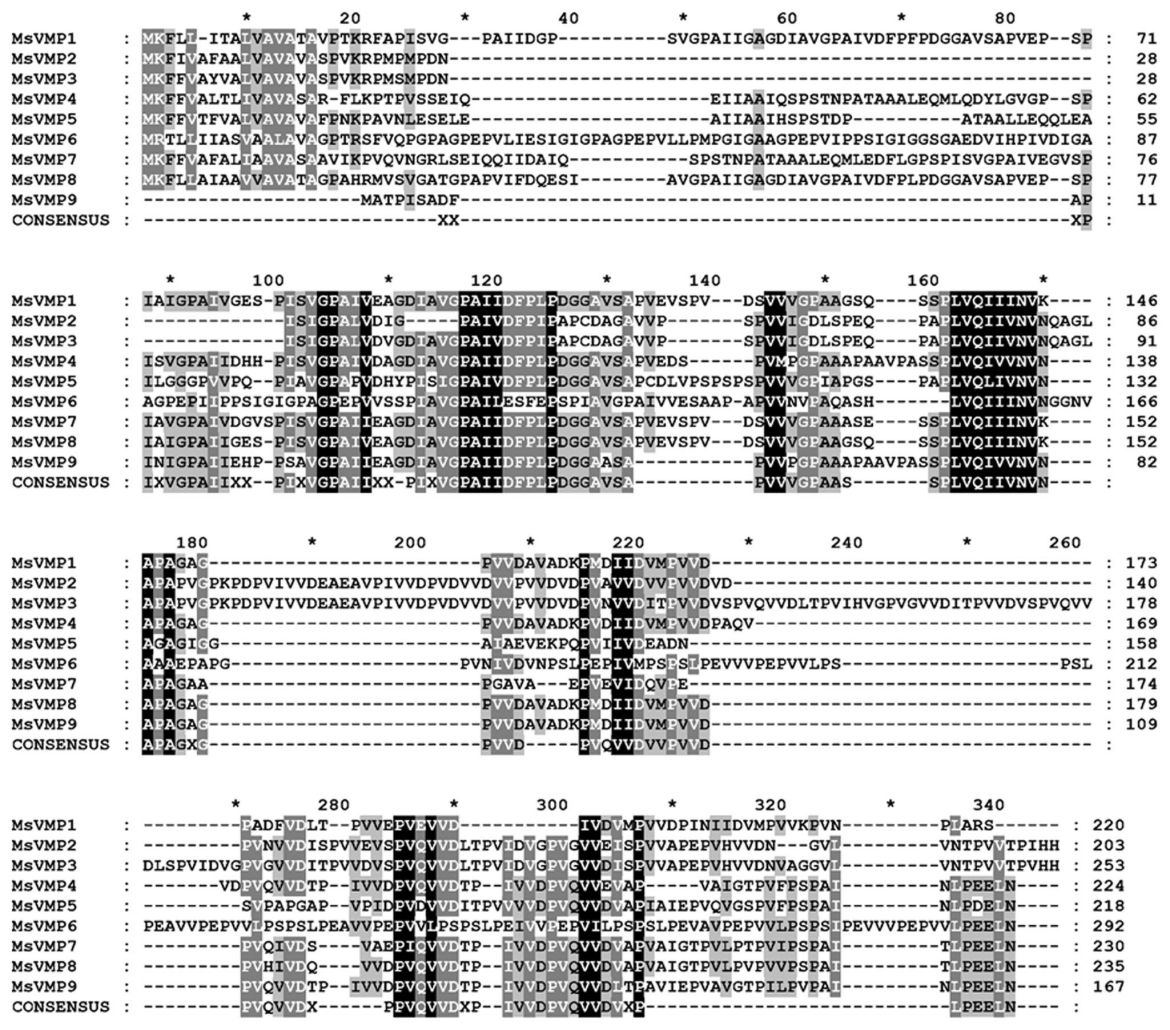
ClustalW alignment of valine-rich midgut proteins from *M. sexta* (MsVmps). Highly conserved or identical amino acids are highlighted with light grey, grey or black shadings. The consensus sequence is given below. The accession numbers are as follows: MsVmp1 (Msex012254-PA), MsVmp2 (central part of Msex012257-PA), MsVmp3 (C-terminal part of Msex012257-PA), MsVmp4 (N-terminal part of Msex012261-PA), MsVmp5 (N-terminal part of Msex012257-PA), MsVmp6 (Msex011100-PA), MsVmp7 (C-terminal part of Msex012261-PA), MsVmp8 (Msex012260-PA), and MsVmp (Msex012262-PA).

To identify proteins that are associated with the PM, we performed an immunoscreening using a midgut λ-ZapII cDNA library from *M. sexta* and polyclonal anti-PM antibodies that we have generated against the components of purified PM from larval midguts (see Material and Methods). From this screening, we obtained ten phage-clones, which were still immune-reactive after two rounds of re-screening. The cDNA inserts from these plaques were sequenced after *in vivo* excision of the corresponding pBluescript (SK-) plasmids. A tblastn database search revealed positive hits for cDNAs encoding proteins that are typically found in the lepidopteran midgut, such as trypsin, chymotrypsin and β-glycosidase (Table 1). Most strikingly, three of the identified cDNA sequences showed similarities to putative cuticle proteins from *Bombyx mori*. A tblastn search of the recently sequenced *Manduca sexta* genome (assembly v1.0) revealed six additional genes with sequence similarities to these three identified cDNAs. These *Manduca* genes encode members of a well-defined family of nine proteins. We termed these proteins *M. sexta* valine-rich midgut proteins 1-9 (MsVmp1-9) due to the high percentage of valine residues (19-31%, Table 2). Besides this high valine content, MsVmps are also characterized by a higher than normal content of proline residues, but they are highly deficient in histidine, cysteine, tryptophan and tyrosine residues (Figure S1, supporting information). The theoretical molecular masses of the proteins range between 16.4 and 28.8 kDa (including the signal peptides), and the isoelectric points between 3.3 and 3.8 (Table 2). They all contain several xPA(V/I)xx motifs in the central region and the highly conserved sequence (S/A)PLVQIIV(N/K) (Figure 1). In contrast to PM proteins (PMPs) and invertebrate intestinal mucins, they lack chitin-binding domains belonging to the CBM 14 family, which have six conserved cysteines with a characteristic spacing between these residues. Due to the high content of hydrophobic amino acids (GRAVY indices: 0.64-0.93, Table 2), we analysed these sequences for the presence of putative transmembrane helices. In none of the MsVmps, however, transmembrane helices were computationally predicted by TMHMM 2.0. All MsVmps contain signal peptides with predicted cleavage sites at amino acid positions 15 for Vmp1, position 16 for Vmp2, -3, -5, -6 and -8, position 20 for Vmp4 and -9 and position 24 for Vmp7. Moreover, Vmp2, 3 and 6 have potential single *N*-glycosylation sites, and all Vmps except Vmp9 have multiple *O*-glycosylation sites (Table 2).

**Table 1:** Results of a tblastn search using the predicted protein sequences obtained from the immunoscreening.

**Accession No**.	**Description**
**ACY06932.1**	similar to hypothetical cuticle protein CPH37 from *Bombyx mori*
**ACY06933.1**	similar to hypothetical cuticle protein CPH38 from *Bombyx mori*
**AEK21795.1**	similar to hypothetical cuticle protein CPH45 from *Bombyx mori*
**L16807.1**	*Manduca sexta* alkaline trypsin
**M77754.1**	*Manduca sexta* fatty acid binding protein 1
**AM690449.1**	*Manduca sexta* chymotrypsinogen-like protein 4 (Ctlp4)
**AAC06038.1**	similar to β-glucosidase from *Spodoptera frugiperda*
**ABD36319.1**	similar to cytosolic malate dehydrogenase from *Bombyx mori*
**AJ519536.1**	*Manduca sexta* mRNA for non-muscle actin (*act3*)
**EU286785.1**	*Manduca sexta* mitochondrion, rRNA large ribosomal subunit
**U09843.1**	*Manduca sexta* cytochrome c oxidase subunit 1 mRNA, mitochondrial

**Table 2:** Protein properties of unprocessed MsVmps.

**Protein**	**Acc. No.**	**Length(aa)**	**Mr (kDa)**	**pI**	**SignalP(aa pos.)**	**Signal peptide**	***N*-/*O*-glycosylation sites**	**GRAVY index**	**V/P content(%)**
**Vmp1**	Msex012254-PA	220	21.7	3.8	15|16	MKFLLITALVAVATA	n.p./S35,S82	0.85	19.1/15.0
**Vmp2**	Msex012257-PA	203	20.5	3.8	16|17	MKFIVAFAALVAVAVA	N28/S59,T199	0.90	29.6/16.7
**Vmp3**	Msex012257-PA	253	25.5	3.7	16|17	MKFFVAYVALVAVAVA	N28/S64,T249	0.93	31.2/15.4
**Vmp4**	Msex012261-PA	224	22.5	3.7	20|21	MKFFVALTLIVAVASARFLK	n.p./S39,S61	0.68	18.8/15.2
**Vmp5**	Msex012257-PA	218	21.8	3.7	16|17	MKFFVTFVALVAVAVA	n.p./S105,S107,S109,S120	0.65	17.4/17.0
**Vmp6**	Msex011100-PA	292	28.8	3.7	16|17	MRTLLIIASVAALAVA	N182/S21,S126,S193,S209,S225,S241,S257,S273	0.64	16.8/22.9
**Vmp7**	Msex012261-PA	230	22.9	3.6	24|25	MKFFVAFALIAAVASAAVIKPVQV	n.p./S42,S62,S75	0.76	17.4/13.9
**Vmp8**	Msex012260-PA	235	22.9	3.7	16|17	MKFLLAIAAVVAVATA	n.p./S88,S225	0.87	20.0/14.9
**Vmp9**	Msex012262-PA	167	16.4	3.3	20|21	MATPISADFAPINIGPAIIE	n.p./n.p.	0.79	19.2/18.0

**Figure 2:**
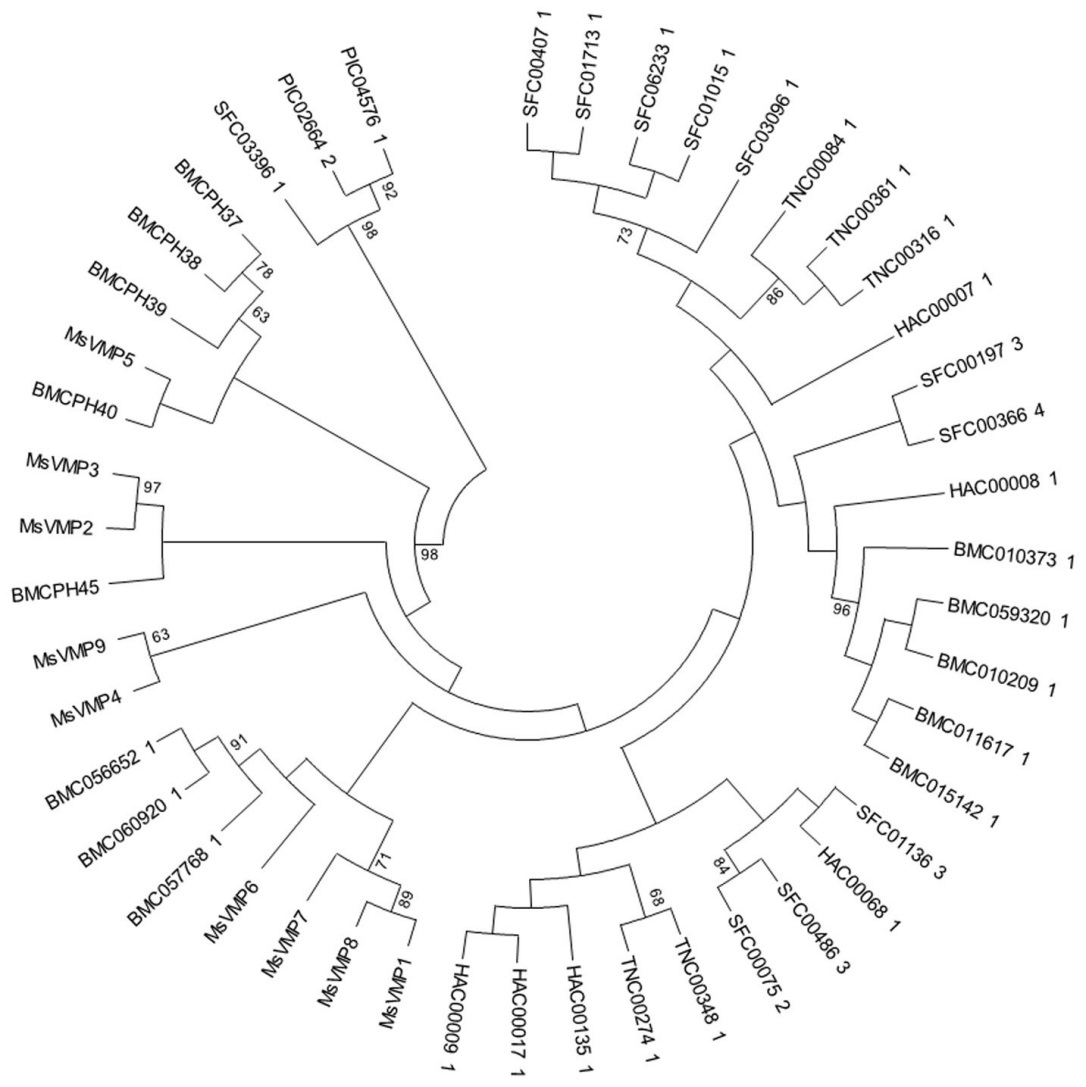
Phylogenetic tree of lepidopteran VMPs. The un-rooted maximum likelihood tree was calculated on the basis of a ClustalW alignment of VMPs from different lepidopteran species. Bootstrap values are given in percentages at the internodes; Accession numbers refer to the entries of the dbEST database of Butterflybase. MS, *Manduca sexta*; BM, *Bombyx mori*; TN, *Trichopulsia* *ni*; HA, *Helicoverpa armigera*; SF, *Spodoptera frugiperda*; PI, *Plodia interpunctella*.

Database searches of various genomes using blastp and a conserved region corresponding to amino acid positions 70-146 of MsVmp1 as query revealed highly significant hits (E values <1e-10) only for lepidopteran species, with the lowest E value of 4e-14 detected for the hypothetical cuticle protein Cph38 from *Bombyx mori* (Table S2, supporting information). Similar results were obtained when using tblastn. No significant hits were obtained for nucleotide or protein sequences of species belonging to any of the other insect orders, or to crustaceans, fishes, reptilians, birds or mammals. The best hits obtained for non-lepidopteran insects had e-values >0.5e-3. Thus, VMPs constitute a family of lepidopteran-specific proteins. Homologs of these genes were found in the genomes of several lepidopteran species including *B. mori* (13 genes) *Spodoptera frugiperda* (11 genes), *Helicoverpa armigera* (6 genes), *Trichoplusia ni* (5 genes), and *Plodia interpunctella* (2 genes). The deduced protein sequences of all of these genes contained xPA(V/I)xx motifs and a region similar to the (S/A)PLVQIIV(N/K) consensus sequence. Phylogenetic analyses of lepidopteran VMPs using the most conserved region (amino acid positions 70 to 146 of MsVmp1) revealed that the *VMP* genes have greatly diversified throughout lepidopteran evolution presumably starting with the duplication of an ancestral gene (Figure 2). Generally, the *B. mori VMP* genes were most closely related to the *MsVMPs*, reflecting the close phylogenetic relatedness between the *Bombycidae* and *Sphingidae* families, to which *B. mori* and *M. sexta* belong, respectively. Homologs of *MsVMPs* were not found in clades of the *Noctuidae* and *Pyralidae* families. Interestingly, five of the 13 *B. mori* genes share greatest similarities with *VMP* genes from *S. frugiperda*, *H. armigera* and *T. ni* (*Noctuidae*). These *B. mori* genes may have been derived from gene duplication events, as they form a monophyletic clade within a group of different lepidopteran species. The finding that *M. sexta* does not have orthologs for any of these five *B. mori* genes further suggests that their common ancestor has been lost in *M. sexta* during evolution of the lepidopteran lineage, because more distantly related species do have orthologous relationships. 

### *VMP* genes are expressed in the larval midgut during feeding stages

**Figure 3:**
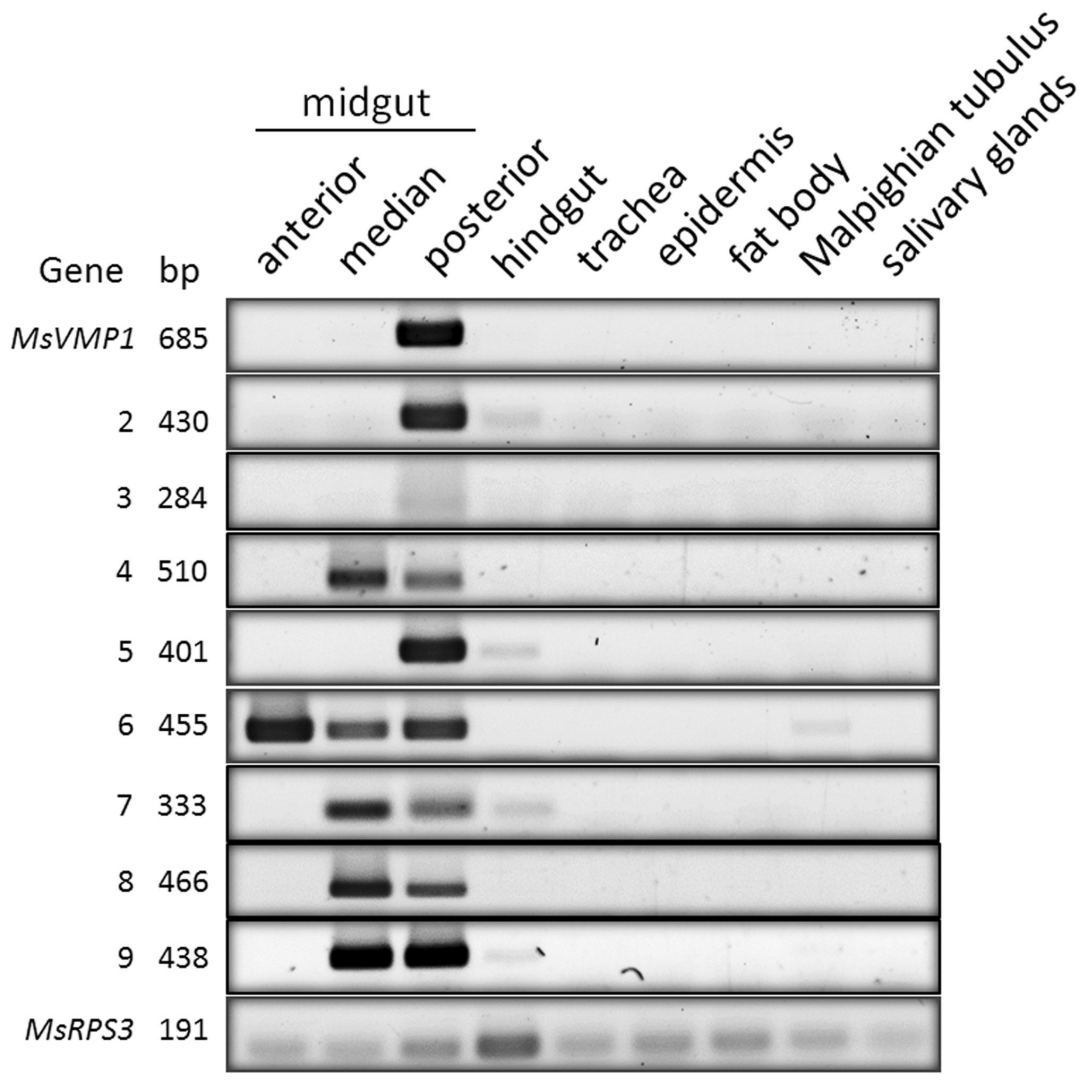
Tissue specific expression of *MsVMP* genes in fifth instar larvae of *M. sexta*. Total RNA was prepared from various tissues and cDNAs were synthesized. RT-PCR was carried out with primers specific to the indicated genes. PCR products of indicated sizes were separated by agarose gel electrophoresis and stained with ethidium bromide. Products for the ribosomal protein MsRpS3 were used as a loading control. Expression was found exclusively in the midgut.

To analyze the expression of *MsVMP* genes in *M. sexta*, we performed RT-PCR using total RNA from different developmental stages and various tissues. While significant *MsVMP* gene expression was observed in larvae of different ages, no transcripts were detectable in eggs, pupae and adults (Figure S2, supporting information). The *MsVMPs* were expressed specifically in larval midgut tissues, while no expression was detected in hindgut, trachea, epidermis, fat body, salivary glands or Malpighian tubules (Figure 3). While the expression of *MsVMP6* was highest in the anterior midgut, expression of *MsVMP4*, *-7*, and *-8* was highest in the median midgut, expression of *MsVMP1*, *-2*, *-3, and -5* was highest in the posterior midgut, and that of *MsVMP9* was equally high in median and posterior midguts. The low levels of transcripts detected for *MsVMP2, -5* and *-7* in the hindgut and for *MsVMP6* in the Malpighian tubules are likely due to contamination during tissue dissection. To test whether the nutritional state or molting affects *MsVMP* expression, we compared mRNA levels in feeding, starving (36 h) and molting larvae (metamorphic molt) by qPCR. All *MsVMP* genes appeared to be expressed in the feeding stage, while molting or starvation leads to a significant drop in the detected mRNA levels (Figure S3, supporting information). Thus, *MsVMP* gene expression appears to be regulated depending on the nutritional state and/or molting, when the larvae also discontinue feeding.

**Figure 4:**
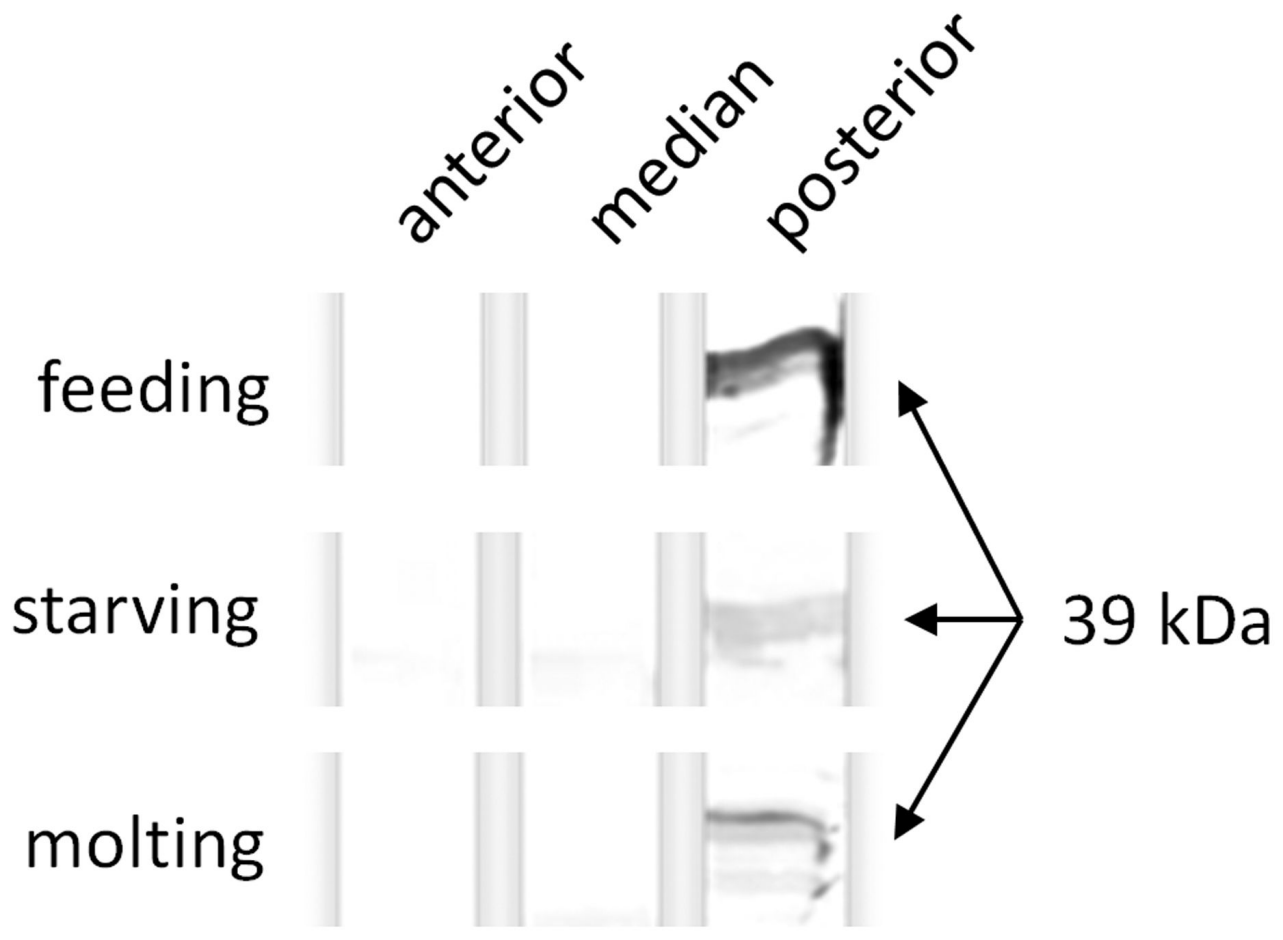
Immunoblotting detects MsVmp1 in the midgut of feeding, starving and molting larvae. Crude protein extracts from the anterior, median and posterior midgut were separated by SDS-PAGE, blotted onto nitrocellulose and stained with anti-VMP1 antibodies. The given molecular mass was estimated using standard proteins of known molecular masses.

As qPCR showed highest transcript levels for *MsVMP1* compared to the other *MsVMP* genes, we analysed this protein in greater detail. To investigate the localization of MsVmp1, we generated polyclonal antibodies to the recombinant protein. For this purpose we expressed Vmp1 in bacteria and purified it by Ni-NTA chromatography. Recombinant MsVmp1 exhibited an anomalous migration behaviour in SDS-PAGE, as it migrates at approximately 50 kDa, more than twice the theoretical molecular mass of 21.7 kDa calculated for MsVmp1 (Figure S4, supporting information). This unusual migration behavior was not influenced by omitting reducing agents during SDS PAGE, denaturing the protein with deionized urea or incubating the sample at room temperature instead of boiling it before loading. Thus, the reason for this unusual migration behavior remains elusive. As we were absolutely sure about the protein identity, we used the recombinant MsVmp1 to immunize guinea pigs. When we tested the obtained anti-Vmp1 antibodies, they reacted strongly with the recombinant protein as well as with two midgut proteins of about 37 and 39 kDa in a crude midgut extract from feeding 5th instar larvae (Figure S4, supporting information). In line with our results from RT-PCR, western blots using protein extracts from the anterior, median and posterior midgut showed that MsVmp1 is highly abundant only in the posterior midgut, and that protein amounts were highest in feeding larvae, and decreased in starving and molting larvae (Figure 4).

### MsVmp1 is modified by *O*-glycosylation

**Figure 5:**
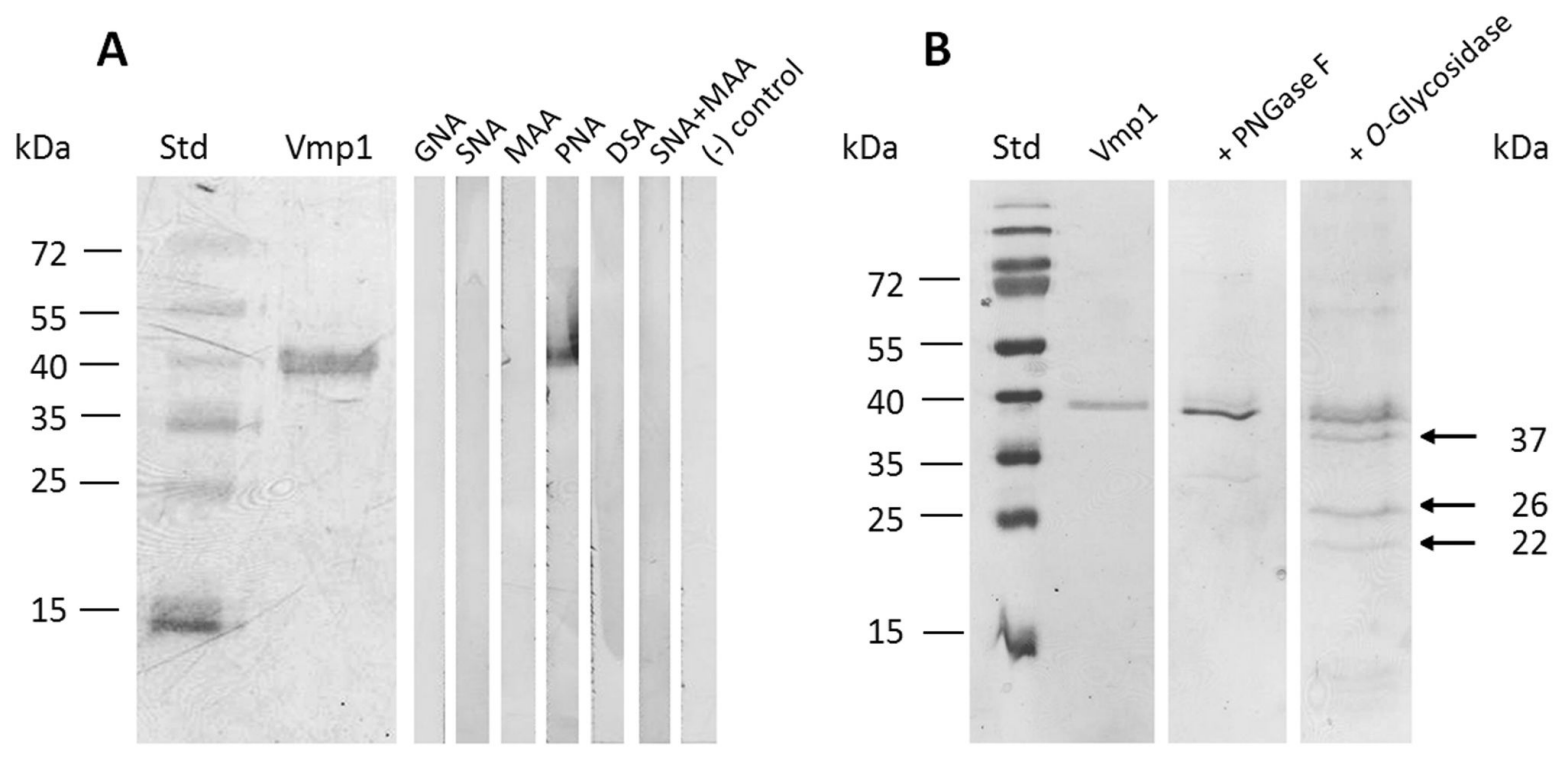
MsVmp1 is extensively *O*-glycosylated. (A) *MsVMP1* was expressed in insect Sf21 cells and purified by Ni-NTA chromatography. After SDS-PAGE, the proteins were transferred to a nitrocellulose membrane and stained with Ponceau Red (left) and subsequently with different lectins (right lanes). GNA, *Galanthus nivalis* agglutinin; PNA, peanut agglutinin; SNA, *Sambucus nigra* agglutinin; DSA, *Datura stramonium* agglutinin; MAA, *Maackia amurensis* agglutinin. (B) For deglycosylation, MsVmp1 (expressed in insect cells) was treated with PNGase F or *O*-glycosidase in the presence of protease inhibitors. The reaction products were separated by SDS PAGE and stained with Coomassie Blue. *Left* *lane*, MsVmp1 input without addition of glycosidase; Middle lane, PNGase F treatment; Right lane, *O*-glycosidase treatment. Std, standard proteins with indicated molecular masses in kDa. Arrows point to reaction intermediates and the terminal deglycosylation product with indicated molecular masses.

The observation that the proteins detected with the anti-Vmp1 antibodies in midgut extracts were significantly larger than the theoretical molecular mass for MsVmp1 suggested that they are modified by glycosylation, significantly increasing their apparent molecular masses in SDS-PAGE. As bacteria lack the eukaryotic glycosylation machinery, we expressed *MsVMP1* in insect cells using a baculoviral expression system, and purified the protein by Ni-NTA chromatography. This recombinant version of MsVmp1 exhibited a molecular mass of about 39 kDa in SDS-PAGE, which was in the range of molecular masses of midgut proteins detected with the anti-Vmp1 antibodies. To test for glycosylation, we performed lectin staining using Western blots of MsVmp1 purified from insect cells. Out of several lectins that we have tested, only peanut agglutinin (PNA) reacted with the recombinant protein indicating that MsVmp1 is *O*-glycosylated, because PNA detects the core disaccharide galactose-β(1–3)-*N*-acetylgalactosamine found in many *O*-glycans. The lack of a positive reaction for *Galanthus nivalis* agglutinin may indicate the absence of high mannose *N*-glycans. The lack of a reaction with *Sambucus nigra*, *Maackia amurensis* and *Datura stramonium* agglutinins may further indicate the absence of sialic acid linked either (2,3) or (2-6) to galactose, and the absence of galactose-β(1–4)-*N*-acetylglucosamine disaccharides (Figure 5). To provide final proof for MsVmp1 being a glycoprotein, we subjected the recombinant protein from insect cells to enzymatic deglycosylation using PNGase F and *O*-glycosidase. While the treatment with PNGase F did not significantly affect the molecular mass of MsVmp1 in SDS PAGE, incubation with *O*-glycosidase resulted in deglycosylation of MsVmp1 with intermediates appearing at 37 kDa and 26 kDa (Figure 5). The smallest protein band corresponds to the completely deglycosylated protein. Its size was about 22 kDa, which is in good agreement with the theoretical molecular mass of 21.7 kDa of MsVmp1. Thus, we provide evidence that MsVMP1 is *O*-glycosylated and that the sugar moieties account for about 17 kDa of the mature MsVmp1 glycoprotein with a molecular mass of about 39 kDa. 

### MsVmp1 is loosely associated with the PM and does not bind chitin

**Figure 6:**
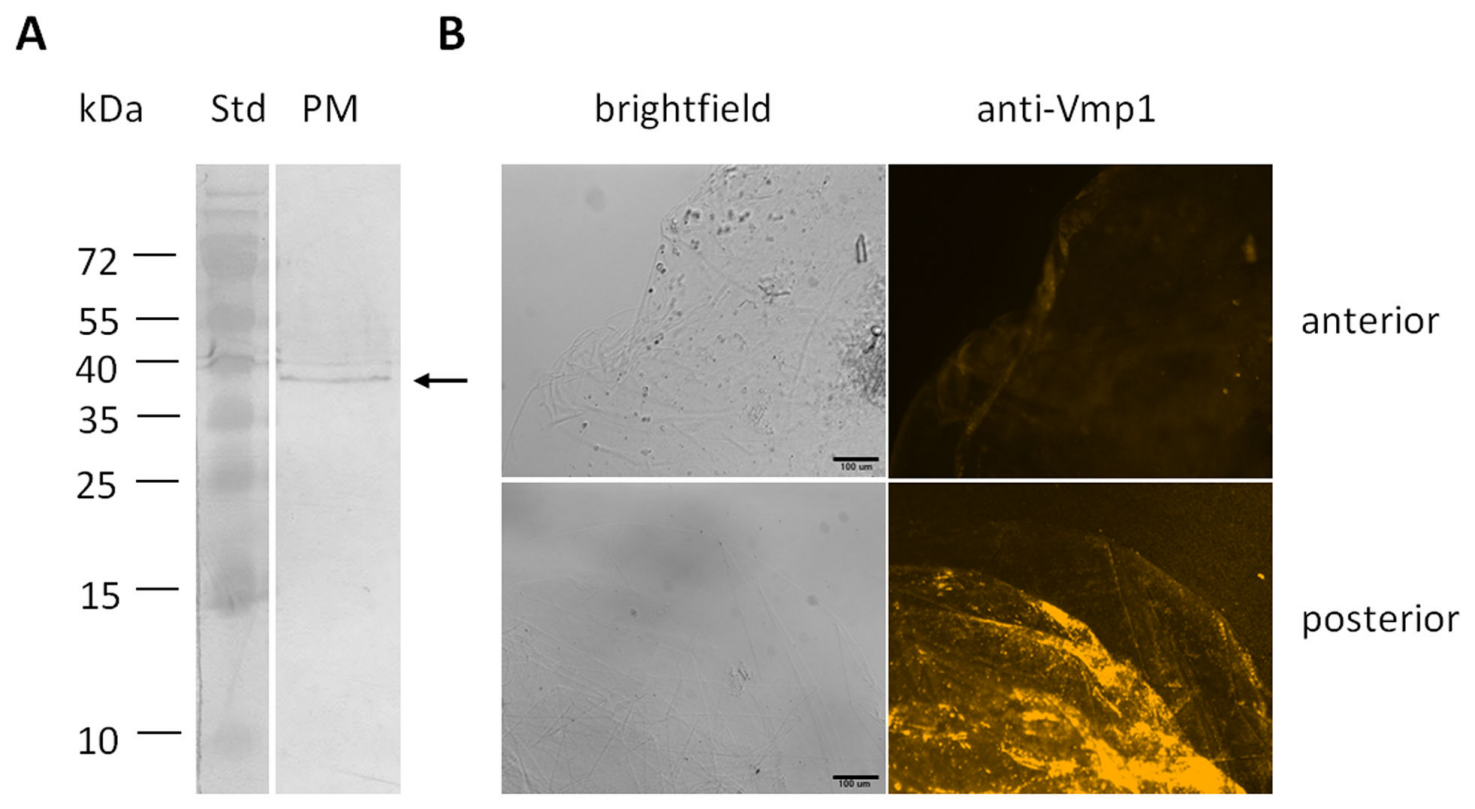
Immunodetection of MsVmp1 in PM preparations from anterior and posterior midguts of feeding larvae. (A) PM proteins were extracted by SDS treatment and separated by SDS-PAGE. Then the proteins were transferred to nitrocellulose and reacted with polyclonal anti-Vmp1 antibodies. Std, standard proteins with molecular masses indicated in kDa. (B) Immunodetection of Vmps using anti-Vmp1 antibodies. The PM preparations from the anterior and posterior parts of the midgut were washed several times with PBS buffer, blocked with bovine serum albumin and stained with the anti-Vmp1 antibodies. Cy3-conjugated anti-guinea pig IgGs were used as secondary antibodies. The PM was transferred to a microscope slide and mounted with Vectashield under a cover slip. The specimens were viewed under a fluorescence microscope using appropriate excitation an emission filters.

MsVmp1 was originally identified in an immunoscreening using polyclonal anti-PM antibodies indicating that the protein is associated with the PM. Indeed, MsVmp1 was detectable with the anti-Vmp1 antibodies in western blots of proteins that were extracted from PM by SDS-treatment (Figure 6A). Also native PM dissected from the posterior midgut and washed only with PBS buffer could be visualized by treatment with anti-Vmp1 antibodies followed by fluorescent secondary antibodies (Figure 6B). In agreement with the expression pattern of *MsVMP1* along the midgut, PM prepared from the anterior midgut did not react with these antibodies. These results suggest that in the posterior midgut, MsVmp1 is associated at least weakly with the PM even though the PM has been washed extensively. The amino acid sequence of MsVmp1, however, does not contain regions similar to known chitin-binding domains, such as CBM14 domains or R&R domains, which have been shown (and/or predicted) to mediate tight binding to chitin fibrils. To test whether MsVmp1 protein is able to bind chitin, we carried out chitin-binding assays using colloidal chitin beads and recombinant MsVmp1 expressed in insect cells. The flow-through after washing the beads contained about as much MsVmp1 as the input material indicating that MsVmp1 did not bind to chitin under these conditions. Consistent with this interpretation, after washing or elution with CFW or SDS, no MsVmp1 was detectable in the collected wash and eluate fractions, even in western blots stained with anti-Vmp1 antibodies (Figure S5, supporting information). 

### MsVmp1 is produced by columnar cells of the posterior midgut of feeding larvae and found in the gut lumen during starvation and molting

**Figure 7:**
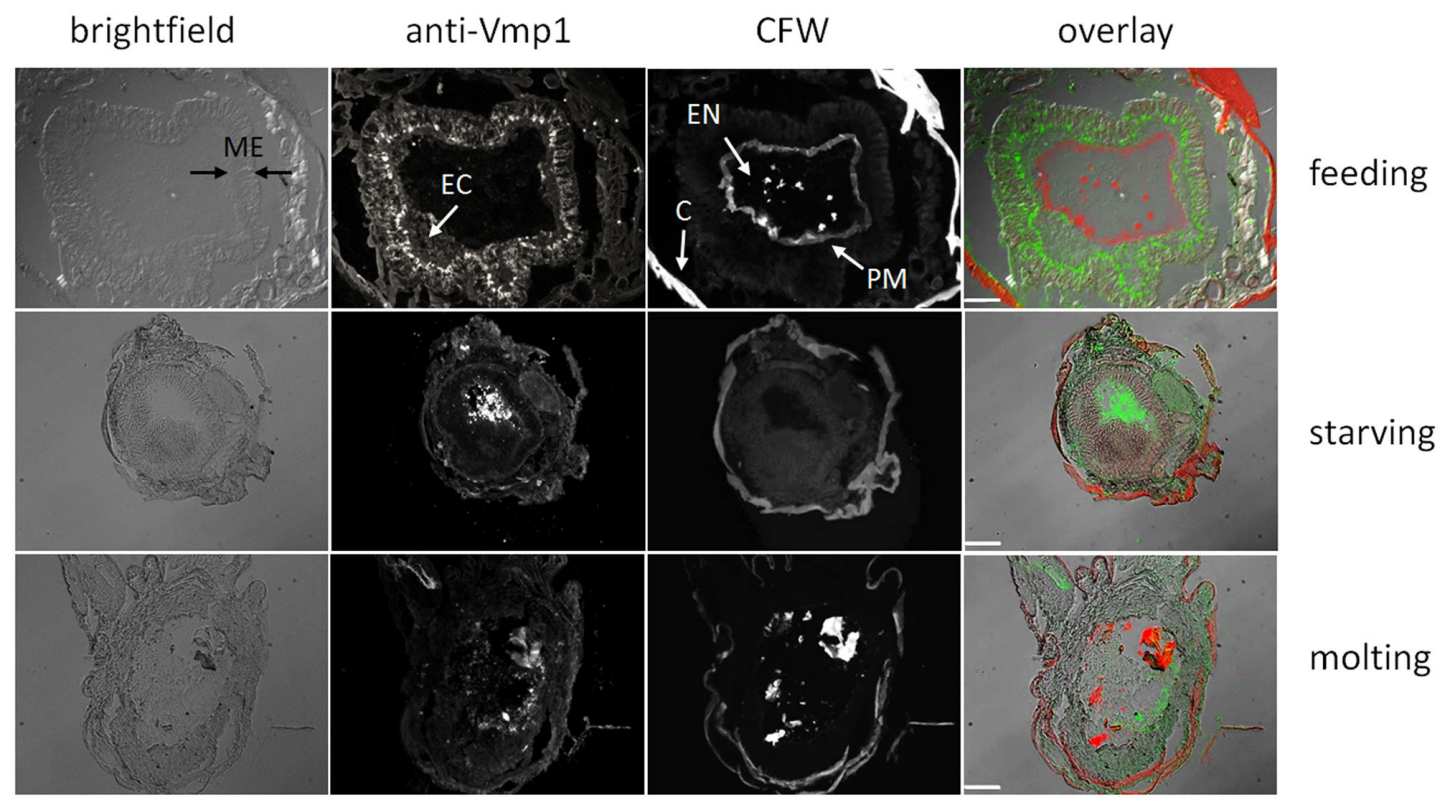
Immunodetection of MsVmp1 in the posterior midgut of *M. sexta* larvae at different physiological conditions. Cryosections of posterior midguts were stained with CFW and immune-labeled with anti-VMP1 antibodies. Primary antibodies were detected with ALEXA 488-conjugated anti-guinea pig IgGs. Brightfield and fluorescence images (for anti-Vmp1 and CFW), and overlays of fluorescence images are shown. Cryosections were obtained from feeding 2^nd^ instar larvae (top), starving 2^nd^ instar larvae (middle) and larvae molting from the 2^nd^ to the 3^rd^ instar. C, cuticle; EC, ectoperitrophic space; EN, endoperitrophic space; PM, peritrophic matrix. Size bar, 100 µm.

To localize MsVmp1 protein, we generated cryosections of the posterior midgut from second instar larvae that were feeding, starving or molting, and stained them with the anti-Vmp1 antibodies. In well fed larvae, MsVmp1 was detected predominantly in the cytosol of columnar cells of the posterior midgut. The signals inside the cells appeared to be in punctate structures (presumably transport vesicles) that are somewhat enriched at the apical site (Figures 7 and S6). The second major cell type in this single-layered gut epithelium, the goblet cells, was essentially free of fluorescence signal (Figure S6, supporting information). In addition, signals were observed in the ectoperitrophic space, indicating secretion of some of the MsVmp1 (Figure 7). In some regions, MsVmp1 was found in the outer region of the PM, as indicated by partial overlap of signals from anti-Vmp1 antibodies and CFW staining in feeding larvae. The endoperitrophic space of the gut lumen was essentially free of MsVmp1 under these conditions. In contrast, in starving and molting larvae, MsVmp1 was not detected in columnar cells, but in the endoperitrophic gut lumen. Apparently, MsVmp1 is produced in columnar cells during the feeding periods, and secreted into the ectoperitrophic space but initially restrained by the PM (Figure 7). To reach the endoperitrophic lumenal space in starving or molting larvae, MsVmp1 has to penetrate the PM, whose permeability appears to be compromised under these conditions compared to the well-fed state. In contrast to feeding larvae, we usually fail to detect the PM in starving or molting larvae in cryosections using CFW, indicating major structural changes of the PM and/or turnover. During periods of starvation or molting, we typically observe macroscopic swelling of the PM, which additionally turns into a highly fragile structure. 

## Discussion

Cuticular proteins have been divided into several families based on the presence of conserved sequence motifs [15]. They include CPR [16], CPF/CPFL [17], Tweedle [18] and CPG [19]. In the present study, we describe a lepidopteran-specific gene family, which encodes valine-rich proteins that are secreted into the midgut lumen. The newly identified MsVmps are most closely related to Cph37-40 and Cph45 of *B. mori* that have been annotated previously as cuticular proteins hypothetical (Cph family), which consists of more than 40 members belonging to different families of cuticular protein [20]. Neither Cph37-40 nor the Cph45 proteins from *B. mori* fit any sequence criteria defining one of the previously described cuticle protein families. Hence the question arises, whether the Cphs from *B. mori*, at least those that share sequence similarities with the MsVmps, are indeed associated with cuticular functions. The finding that genes that were initially associated with cuticular functions have been finally proven to have functions in the midgut, and *vice versa* indicates that excessive reliance on sequence similarities alone can lead to incorrect interpretations of the biological functions of these proteins. For instance, the drosocrystallin protein, which contains the RR-1 variant of the Rebers & Riddiford type chitin-binding domain typically found in cuticle proteins, has recently been shown to be essential for the PM’s function as an anti-infectious barrier [21]. Moreover, many genes that encode proteins with one or more CBM14 domains (which are also termed peritrophin A domains because they were originally described for PM proteins) have been shown to be exclusively expressed in cuticle-forming tissues. These proteins have been renamed recently as “cuticular proteins analogous to peritrophins” (CPAPs) and shown to be required for the structural integrity of cuticular material and/or for molting [13].

The expression data provided in this study demonstrate that all *MsVMPs* are exclusively expressed in the midgut. Therefore, it is likely that even the *B. mori CPH* genes, in particular *CPH37-40*, *CPH45* as well as *BMC056652_1*, *BMC060920_1* and BMC057768_1, which share the highest sequence similarities with *MsVMPs*, may be expressed in the midgut. Indeed, a *CPH*-like gene (*CPH45*) has been shown recently to be a midgut specific gene [22]. RNAi to suppress the expression of this gene delayed larval development, led to abnormal midgut morphology and resulted in decreased sex-specific tolerance to infections with cytoplasmic polyhedrosis viruses [22]. Th*e B. mori* gene, *CPH45*, is most closely related to *MsVMP2* and *MsVMP3* (Figure 2). Comparing the expression patterns of these two genes reveals striking similarities. Like *CPH45*, *MsVMP2/3* expression is up-regulated during feeding periods and down-regulated during starvation and molting (compare Figure S3 and [22]). Based on sequence similarities and expression data, we propose that *MsVMP2* or *MsVMP3* might be functional orthologs of the *B. mori CPH45* gene. Sex-specific differences in the expression of *CPH45* were observed with significantly higher mRNA levels in male silkworms in most stages. Therefore, it has been suggested that *CPH45* gene has antiviral functions in the midgut of specifically male silkworms [22]. It will be interesting to see, whether *MsVMP2/3* also show sex-specific expression. Future RNAi studies in *M. sexta* may provide functional insight into the role of MsVmp2/3 and the other MsVmps, although RNAi in *M. sexta* could be less efficient than that in *B. mori* [23]. 

The absence of sequence similarities between MsVmps and gut proteins that are known to associate tightly with the chitin portion of the PM (such as PMPs, insect intestinal mucins, some chitinases and chitin deacetylases with CBDs) suggests that MsVmps are not integral constituents of the PM. In agreement with this notion, we could not detect chitin-binding activity for the purified, recombinant MsVmp1, which was expressed in insect cells to allow post-translational modifications. Although we can detect this protein in preparations of the PM, MsVmp1 seems to be associated with the PM only transiently and loosely. This conclusion is based on immunohistochemistry, which indicates that only a minor fraction of this protein is associated with the outer region of the PM in feeding larvae, even though the protein is most abundant in feeding insects (Figure 4). Thus, MsVmp1 may be nonspecifically trapped by the porous meshwork of the outer PM. Alternatively, Vmps may interact loosely with PMPs possessing chitin binding domains. Interestingly, the PM seems to undergo significant changes in its structure and protein composition in starving or molting larvae [24], which may account for the altered permeability and the movement of MsVmp1 from the ectoperitrophic space into the endoperitrophic space of the gut lumen. MsVmp1 is clearly a secretory protein, due to the presence of a signal peptide and the absence of any membrane-anchoring sequences. Correspondingly, it is found in the cytosol of columnar cells and in the gut lumen but not at plasma membranes of columnar cells, because the brush border does not react with the anti-Vmp1 antibodies (Figures 7 and S6). The functional significance of the accumulation of MsVmp1 specifically in the gut lumen of starving larvae remains unclear. As suggested for Cph45 from *B. mori*, MsVmp1 may participate in maintenance of the integrity of the midgut and/or have a function in midgut immunity [22]. To test for possible antimicrobial activities, we performed growth tests in the presence and absence of recombinant Vmp1. Neither gram (+) *Bacillus subtilis*, gram (-) *Escherichia coli*, nor the fungal pathogen *Beauveria bassiana* exhibited inhibition of growth rates by MsVmp1 at a concentration of 2 µg/ml, suggesting that MsVmp1 has no antimicrobial activity at this concentration (Figure S7, supporting information). However, the possibility that MsVmp1 may have antiviral activities needs to be tested in future experiments. MsVmps are characterized by short glycine-rich and alanine/proline/valine-rich repeats, which are frequently found in intrinsically unstructured proteins (IUP). IUPs can have important biological functions when they adopt a structural folding induced by interactions with other protein [25]. Therefore, we performed a yeast two-hybrid screening for potential interaction partners by using a Matchmaker cDNA library of *M. sexta*, which was successfully used to identify a binding partner of the midgut-specific chitin synthase MsChs2 [26]. However, we could not detect positive interactions under stringent selection conditions when we used *MsVMP1*-cDNA as bait. 

MsVmp1 has a theoretical molecular mass of 21.7 kDa but the purified recombinant protein expressed in insect cells migrates with an apparent mobility of about 39 kDa in SDS-PAGE. Similarly, the midgut proteins detected with the anti-Vmp1 antibodies migrated at about the same molecular mass. Therefore, we speculated that glycosylation of this protein could increase the molecular mass and result in different glycoforms of MsVmp1. Further, many attempts to dissociate non-covalent interactions such as dimerization or stable association with other macromolecules did not alter the migration behaviour. Insect midgut proteins, such as intestinal mucins, are frequently modified by extensive glycosylation [27]. In the case of MsVmp1, we identified two potential *O*-glycosylation sites (Table 2). Using lectin stainings and enzymatic deglycosylation, we could demonstrate that extensive *O*-glycosylation accounts for the shift in the molecular mass of MsVmp1. *O*-glycosylation is a widespread and complex post-translational modification of proteins found not only in animals, but also in higher plants [28]. During *O*-glycosylation, long filamentous polysaccharides are added to threonine and/or serine residues in the Golgi apparatus. The sugar backbone frequently contains galactose and/or *N*-acetylglucosamine that are linked by different types of bonding. *O*-glycosylation may strongly influence the properties and functions of MsVmp1. Despite increasing insights into the biosynthesis of glycoproteins and *O*-glycosylation, there is still only limited information on the functional significance of this modification. It is well established that *O*-glycosylation shields the protein core from spontaneous hydrolytic events in the aqueous environment and confers resistance against proteases. *O*-glycans may also influence the conformation of the proteins to which they are attached [28]. In some cases, a role in the defense against pathogenic microorganisms has been suggested for *O-*glycans by providing multivalent binding sites for bacterial adhesins that lead to the entrapment of bacteria within the fibrous matrix [29]. Finally, recognition of *O*-linked glycans by soluble lectins or membrane-bound carbohydrate-binding receptors is also involved in signaling processes in innate immunity [30]. It will be a challenge for future work to elucidate the role of *O*-glycosylation in MsVmp1 and to uncover the physiological functions of the other members of this unusual protein family. 

## Materials and Methods

### Chemicals

Routine chemicals were purchased in P. A. quality from Sigma-Aldrich (Hamburg, Germany). Primers (Tab. S1, supporting information) were synthesized by Eurofins MWG Operon (Ebersberg, Germany). 

### Experimental animals

*Manduca sexta* larvae were reared under long-day conditions (16 h of light at 27 °C) using a synthetic diet modified according to [31].

### Immunoscreening

Immunoscreening of a λ-Zap-cDNA library from the posterior midgut of *M. sexta* was performed as described previously [32]. About 1 x 10^5^ plaque-forming units were screened using a 1:150 dilution of the polyclonal anti-PM antiserum (see below), and a 1:10,000 dilution of the secondary anti-guinea-pig antibody (Sigma-Aldrich). Following three re-screening steps with stepwise reduced phage titers, several antibody-positive clones were isolated. The corresponding cDNAs were sub-cloned by *in vivo* excision into pBlueskript (SK-) according to [33]. 

### Gene expression studies

Total RNA was isolated by using the TRIZOL reagent according to the manufacture’s protocol (Invitrogen, Carlsbad, CA, USA). cDNA synthesis was performed with Superscript III first strand synthesis system (Invitrogen). The expression profile for each gene was determined with pairs of gene-specific primers (Tab. S1, supporting information). The constitutively expressed house-keeping gene encoding the *M. sexta* ribosomal protein S3 (MsRpS3) was used as a loading control. The PCR reactions were carried out at the following conditions: denaturation at 92 °C for 30 s, annealing at 57 °C for 45 s and elongation at 72 °C for 1 min for 30 cycles. PCR products were separated by agarose gel electrophoresis and stained with ethidium bromide. qPCR was carried out as described previously [34].

### Heterologous expression of recombinant proteins

For antigen preparation, the cDNA encoding MsVmp1 was expressed in *E. coli* BL21 Codon plus (DE3)-RIL. The *MsVMP1*-cDNA was amplified using the primers pet29b-VMP1-F and pET29b-VMP1-R (Tab. S1, supporting information). The resulting PCR product was restricted with *BamH*I and *EcoR*I, and ligated into the linearized pET29b vector (Novagen), which encodes a carboxy-terminal hexa-His-Tag for protein purification. Expression in *E. coli* BL21 cells was induced by adding 1 mM IPTG and continued for 6 h at RT. For baculoviral expression, the *MsVMP1*-cDNA was amplified using the primers pFBD-VMP1-F and pFBD-VMP1-R (Tab. S1, supporting information). The resulting PCR product included a sequence encoding a carboxyterminal octa-His-Tag. The *MsVMP1*-cDNA was ligated into the pFast-Bac-Dual vector (pFBD, Invitrogen), which additionally contained a cDNA sequence encoding the enhanced green fluorescence protein (EGFP) under the control of the p10 promoter [35]. The resulting pFBD-*MsVMP1* vector was used to transform DH10Bac cells, to allow transposition into the baculovirus bacmid. The bacmid DNA was isolated according to the Invitrogen protocol, and used for the transfection of *Spodoptera frugiperda* (Sf21) cells to generate a P1 viral stock. After 72 h, budded viruses were harvested from the cell culture medium. A baculoviral stock was used to generate a high-titer P2 stock. Subsequently, the P2 stock was used to infect Sf21 cells, which were assayed for expression of the recombinant protein starting after 3 days. The cells were harvested 5 days after infection, when significant EGFP expression could be observed with a fluorescence microscope (Zeiss Discovery V8.0). Affinity purification of proteins by Ni-NTA agarose column was performed according to the manufacturer’s protocol (His·Bind^®^ Kit, Novagen/Merck, Darmstadt, Germany).

### Generation of antibodies

To obtain anti-PM antibodies, the PM was isolated from ten 5^th^ instar larvae of *M. sexta*, and washed several times with PBS (20 mM KH_2_PO4, 20 mM NaH_2_PO_4_, 0.15 M NaCl, pH 7.4) until they appeared completely transparent and devoid of any food material. The washed PM was frozen in liquid nitrogen, thoroughly ground and resuspended in 1 ml deionized H_2_O, and finally used to immunize two guinea pigs (Charles River Inc., Sulzfeld, Germany). To generate anti-Vmp1 antibodies, recombinant MsVmp1 purified from *E. coli* BL21 cell lysates was used for the immunization of two guinea pigs (Charles River, Sulzfeld, Germany), whose pre-immune sera did not cross-react with midgut proteins from *Manduca* 5^th^ instar larvae. Four booster injections each involving 150 µg protein were administered. After the fourth booster injection, the antisera were collected and purified by protein-A affinity chromatography.

### Cryosectioning and immunohistochemistry

The tissue samples were fixed overnight at 4 °C in a solution of 4 % (w/v) formaldehyde in PBS. After removing the fixation solution, the tissue was washed for 3 x 10 min in 20 mM PBS. Cryosectioning and immunohistochemistry were performed as described previously [36] [9]. For localization of MsVmp1, the cryosections were treated with a 1:100 dilution of the anti-Vmp1 antibodies. Visualization of the primary antibodies was performed with ALEXA Fluor 488 goat anti-guinea pig antibodies at a 1:200 dilution (Invitrogen). To test for unspecific binding of the secondary antibodies, control reactions were carried out without primary antibodies. Next, the sections were rinsed 3 times for 5 min with PBS. For Calcofluor white (CFW, Sigma) staining, the sections were incubated in a 0.01% (w/v) solution for 60 min at room temperature and washed three times with PBS. Finally, the specimens were covered with Vectashield Mounting Medium (Vector Laboratories Inc, Burlingame), the coverslips were sealed with Fixo gum and the sections were viewed under an Olympus IX70 fluorescence microscope using appropriate filter sets.

### SDS-Page, immuno- and lectin-labeling

Crude protein extracts and different larval tissues were prepared as described previously [37]. SDS-PAGE was carried out essentially as described by 38, and semidry electroblotting (Biorad) onto nitrocellulose membranes (Millipore, Schwalbach, Germany) was performed according to [39], modified by the addition of 20% (v/v) methanol. Membranes were stained with 0.02% (v/v) Ponceau S (Sigma-Aldrich). Immunostaining was performed as described previously [40]. Polyclonal anti-VMP1 antibodies (1:100) were used as primary antibodies, and anti-guinea pig antibodies conjugated to alkaline phosphatase were used as secondary antibodies (1:10,000; Sigma-Aldrich). Lectin binding was examined with the DIG Glycan Differentiation Kit (Roche Diagnostics, Grenzach-Wyhlen, Germany) following the manufacturer’s recommendations. 

### Glycosidase treatment

Peptide *N*-Glycosidase F (PNGase F, Roche) cleaves asparagine linked oligosaccharide from glycoproteins. For deglycosylation, 10 µg recombinant MsVmp1 was treated with 4 U PNGase F in a total volume of 100 µl using a buffer consisting of 20 mM sodium phosphate, pH 7.2. MsVmp1 was denatured by boiling it for 2 min in the presence of 0.1% (w/v) SDS. Next, a 10-fold molar excess of Triton-X-100 (compared to SDS) was added to the sample to avoid inactivation of PNGase F by SDS. Subsequently, PNGase F was added and the reaction mixture was incubated for 6 h at 37 °C. *O*-glycosidase cleaves unsubstituted gal-β(1-3)galNAc-alpha disaccharides attached to serine or threonine residues of glycoproteins or glycopeptides. Treatment of Vmp1 with *O*-glycosidase was conducted by incubating 2 µg of MsVmp1 in 50 µL of a 0.02 M sodium phosphate buffer (pH 6.0) containing 0,1% (w/v) SDS, 1% (v/v) Non-ident P-40, 1 mM phenylmethylsulfonylfluoride and 2 mU *O*-glycosidase (Roche Diagnostics, Rotkreuz, Swiss) at 37 °C for 24 h. Following deglycosylation, the samples were analyzed by SDS-PAGE.

### Other methods

Protein concentrations were determined by the Amido Black method [41]. The chitin binding assay was performed using a modified protocol reported by [42]. In this assay, 50 µg purified recombinant MsVmp1 protein purified from insect cell lysates and diluted in 1 ml of a binding buffer containing 0.5 M NaCl, 0.05% (w/v) Triton-X100, 10 mM Tris-HCl (pH 7) and a protease inhibitor cocktail (Roche) was bound overnight at 4°C to 1 ml of washed colloidal chitin beads (New England Biolabs, Ipswich, MA, USA). The suspension was transferred to a 2 ml plastic column, and the beads were washed with 3 column volumes binding buffer and 1 column volvolume 5 mM NaCl, 5 mM Tris-HCl (pH 7). Elution was carried out either with 1% CFW or 2% SDS (each in PBS buffer). Flow-through, wash and elution fractions were collected, and the beads were boiled with 1% SDS. Each fraction was subjected to SDS PAGE. The proteins were either stained with Coomassie blue or transferred to a nitrocellulose membrane and immune-labeled with anti-VMP1 antibodies. Nucleotide sequencing was done by Seqlab GmbH, Göttingen, Germany. Prediction of signal peptide cleavage sites was performed with SignalP 4.0 [43]. GRAVY indices were determined according to [44]. Scanning for potential *N*- and *O*-glycosylation sites was done with the NetNGlyc 1.0 and DictyOGlyc 1.1 Servers (http://www.cbs.dtu.dk/services/NetNGlyc and ~DictyOGlyc). Transmembrane helices were predicted with the TMHMM 2.0 server [45]. For phylogenetic analyses, the highly conserved region of MsVmp1 (amino acid positions 70-146) was used for “blastn”, “blastp” and “tblastn” searches of different protein and nucleotide databases (www.ncbi.nlm.nih.gov/blast, www.butterflybase.org, http://butterflybase.ice. mpg.de). Alignment of amino acids sequences was performed with ClustalW (EMBL-EBI), and the maximum-likelihood tree was constructed with the Mega 5.0 software package. 

## Supporting Information

Figure S1 **Amino acid composition of Vmps from *Manduca sexta*.** The proteins contain a strikingly high number of valine and proline residues, and a low number of histidine, cysteine and aromatic amino acid residues.
(TIF)

Figure S2 **Stage specific expression of *MsVMP* genes.** Total RNA was prepared from various eggs, different-aged larvae, pupae and adults, and cDNAs were synthesized. RT-PCR was carried out with primers specific to the indicated genes. PCR products of indicated sizes were separated by agarose gel electrophoresis and stained with ethidium bromide. Products for the ribosomal protein MsRpS3 were used as a loading control. *MsVMP* transcripts were detectable only in larval stages.
(TIF)

Figure S3 **Expression levels of different *MsVMPs* in the posterior midgut at different physiological conditions.** Total RNA was prepared from posterior midguts that were isolated from feeding 5^th^ instar larvae, starving 5^th^ instar larvae and larvae at the metamorphic molt. cDNA was synthesized by reverse transcription. Aliquots corresponding to 50 ng of total RNA were used as templates for qPCR with 42 cycles. mRNA amounts were determined on the basis of the C_T_-values (mean values ±S.E., *n* = 3). Normalization was performed with reactions amplifying the cDNA of the ribosomal protein S3 from *M. sexta* (MsRpS3; embl accession U12708). Relative expression levels are given in percent of the level reached at the feeding larval stage.
(TIF)

Figure S4 **Immunoblots to detect MsVmp1 using anti-Vmp1 antibodies.** (A) *MsVMP1* was expressed in *E. coli* BL21 cells, purified by Ni-NTA chromatography and separated by SDS-PAGE. (B) Crude extracts from the posterior midgut of *M. sexta* fifth instar larvae were subjected to SDS-PAGE. The proteins were blotted onto nitrocellulose and reacted with anti-Vmp1 antibodies. Std, standard proteins with indicated molecular masses in kDa.
(TIF)

Figure S5 **Chitin binding assay reveals no interaction between MsVmp1 and colloidal chitin.** Purified, recombinant MsVmp1 (expressed in insect cells) was incubated with chitin beads, transferred to a column, washed and eluted either by SDS or CFW treatment. (A) Aliquots of the resulting flow-through, wash and eluate fractions were separated by SDS-PAGE and stained with Coomassie blue. The smear at around 25 kDa in the eluate fractions is due to the presence of CFW and does not contain proteinaceous material. Specifically, it is not detected by anti-Vmp1 antibodies as shown in the following immunoblot. (B) Aliquots of the fractions were separated by SDS-PAGE, blotted onto nitrocellulose and immune-labeled with anti-VMP1 antibodies. Std, pre-stained standard proteins with indicated molecular masses in kDa.
(TIF)

Figure S6 **Immune-detection of MsVmp1 in the posterior midgut from 5^th^ instar larvae of feeding *M. sexta*.** Left panel, a 20 µm cryosection of the posterior midgut was imaged by DIC microscopy. Right panel, the cryosection was stained with anti-Vmp1 antibodies. The primary antibodies were detected with ALEXA 488-conjugated anti-guinea pig IgGs. Antibody signals were visualized using an inverse fluorescence microscope and appropriate filters for excitation and emission tag. BB, brush border; GC, goblet cell; CC, columnar cell; Size bar, 100 µm.
(TIF)

Figure S7 **Anti-microbial activity assays.** MsVmp1 (expressed in insect cells) was assayed for antimicrobial activity. (A) Disc diffusion assay using 2 µg/ml of MsVmp1 on agar plates inoculated with gram (-) *E. coli* DH5α, gram (+) *B. subtilis,*and the fungus *B. bassiana* at titers yielding confluent growth. As positive controls, the antibiotics Genatmycin and Amphotericin B were used. MsVmp1 did not exhibit antimicrobial activity. (B) Growth tests in the presence and absence (-) of MsVmp1 (2 µg/ml). Gentamycin was used to inhibit the growth of gram (-) *E. coli* and gram (+) *B. subtilis*.
(TIF)

Table S1 **Primer sequences.**
(DOCX)

Table S2 **Blastp search for *MsVmp* orthologs in different insect orders and other taxonomic groups.** The accession numbers of proteins revealing the best hits (lowest E values) within the indicated group are given.
(DOCX)
