# Supplementary figures and images for: A Lepidopteran-Specific Gene Family Encoding Valine-Rich Midgut Proteins
Source: PLoS One. 2013 Nov 29;8(11):e82015. doi: 10.1371/journal.pone.0082015 (PMC3843731; doi:10.1371/journal.pone.0082015)

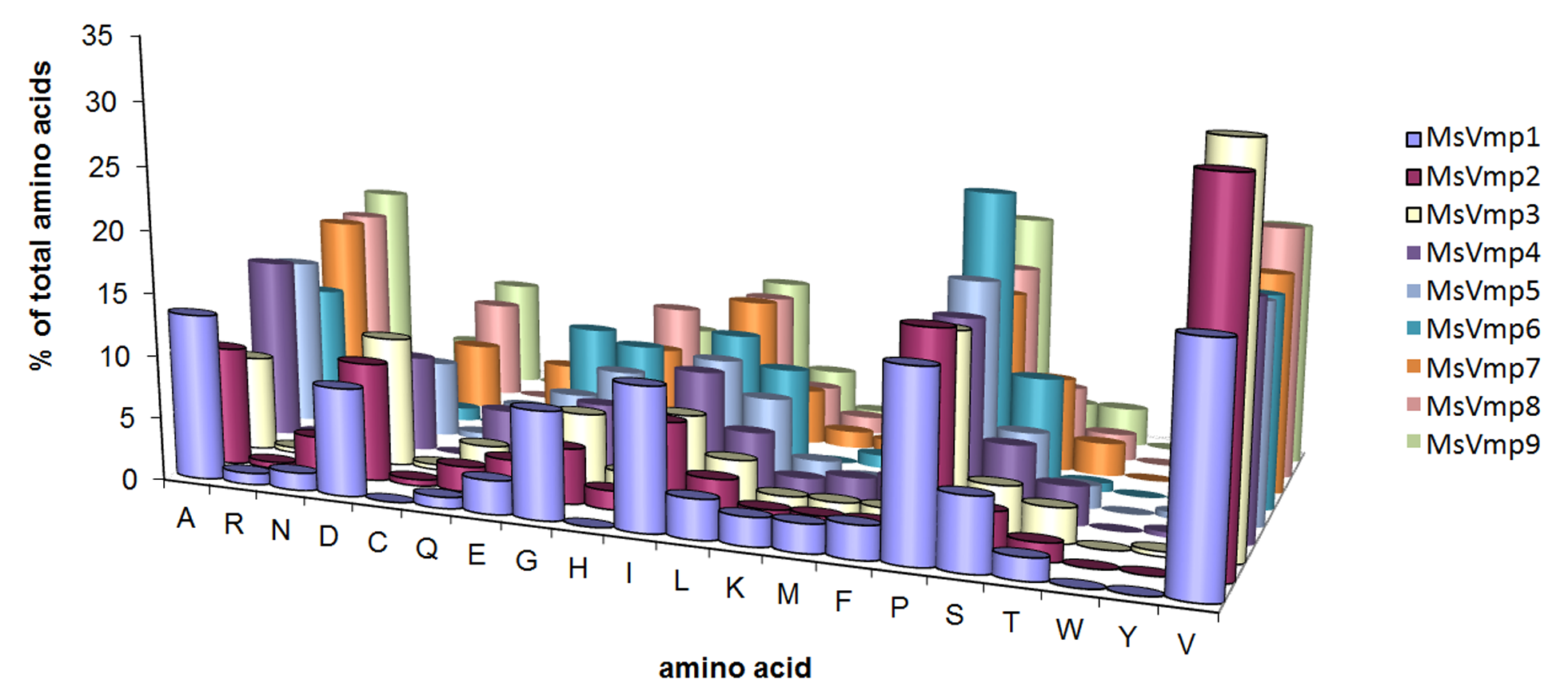

Supplement: Figure S1 — Amino acid composition of Vmps from Manduca sexta. The proteins contain a strikingly high number of valine and proline residues, and a low number of histidine, cysteine and aromatic amino acid residues. (TIF) [file pone.0082015.s001.tif]

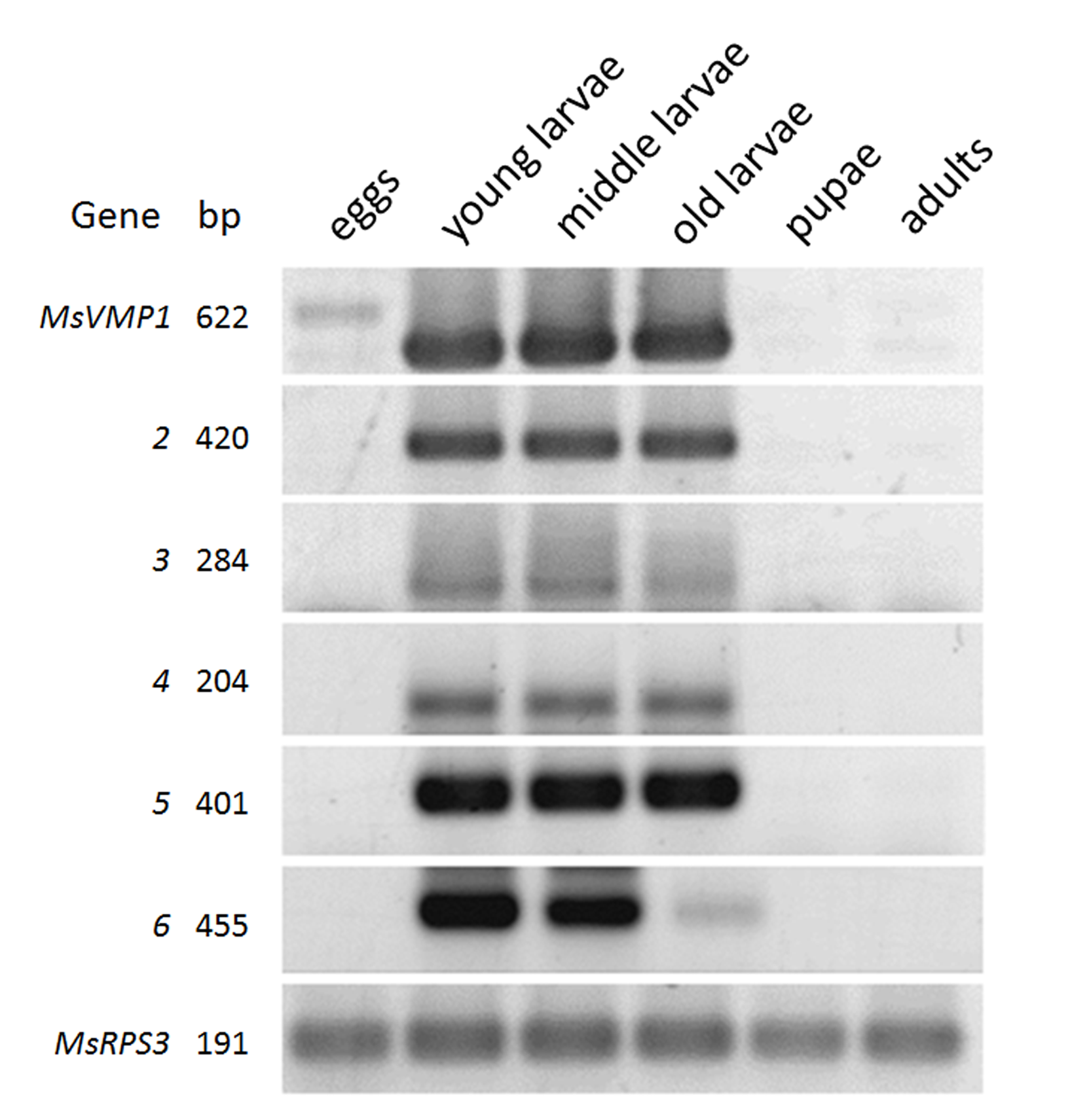

Supplement: Figure S2 — Stage specific expression of MsVMP genes. Total RNA was prepared from various eggs, different-aged larvae, pupae and adults, and cDNAs were synthesized. RT-PCR was carried out with primers specific to the indicated genes. PCR products of indicated sizes were separated by agarose gel electrophoresis and stained with ethidium bromide. Products for the ribosomal protein MsRpS3 were used as a loading control. MsVMP transcripts were detectable only in larval stages. (TIF) [file pone.0082015.s002.tif]

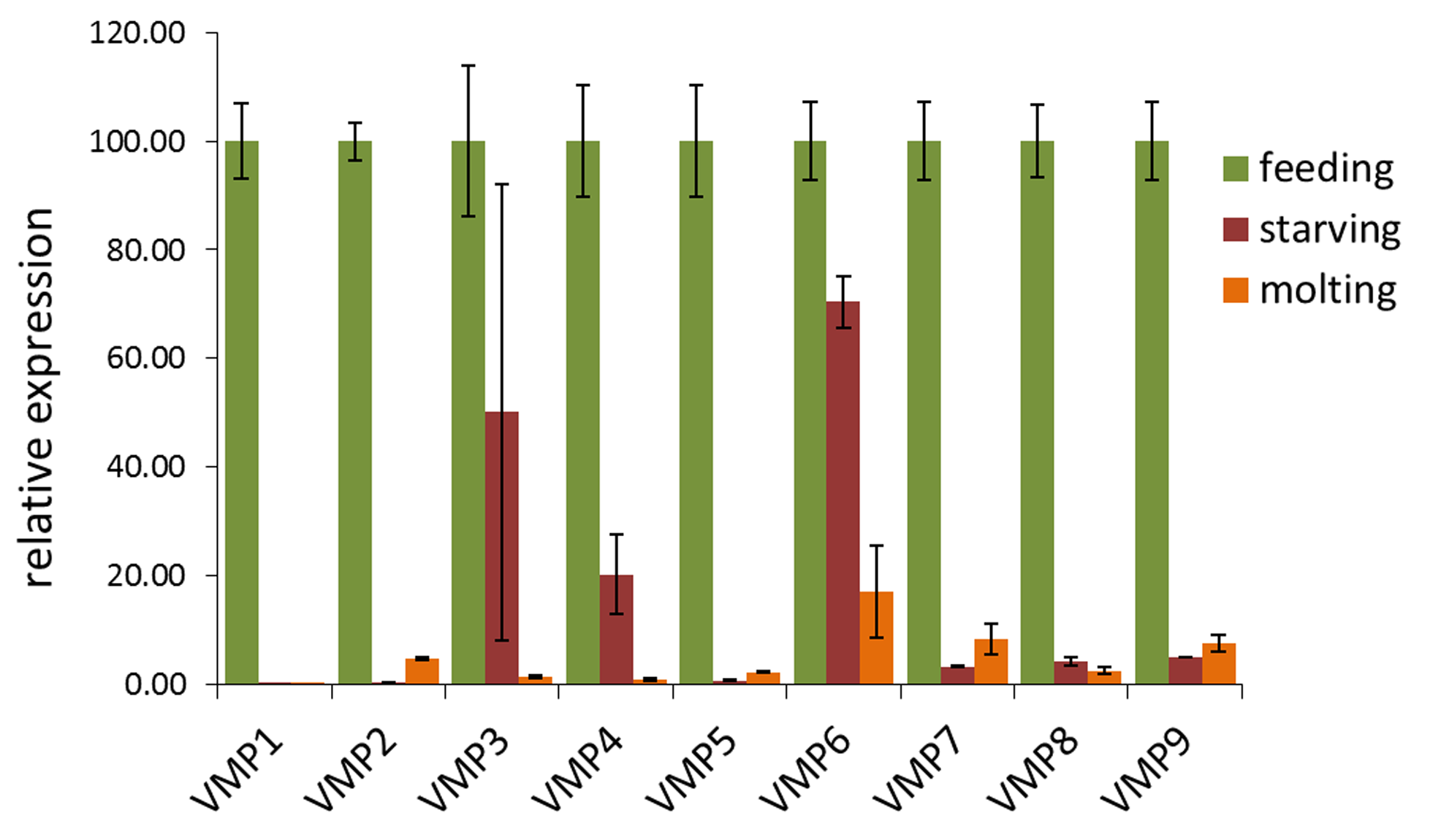

Supplement: Figure S3 — Expression levels of different MsVMPs in the posterior midgut at different physiological conditions. Total RNA was prepared from posterior midguts that were isolated from feeding 5th instar larvae, starving 5th instar larvae and larvae at the metamorphic molt. cDNA was synthesized by reverse transcription. Aliquots corresponding to 50 ng of total RNA were used as templates for qPCR with 42 cycles. mRNA amounts were determined on the basis of the CT-values (mean values ±S.E., n = 3). Normalization was performed with reactions amplifying the cDNA of the ribosomal protein S3 from M. sexta (MsRpS3; embl accession U12708). Relative expression levels are given in percent of the level reached at the feeding larval stage. (TIF) [file pone.0082015.s003.tif]

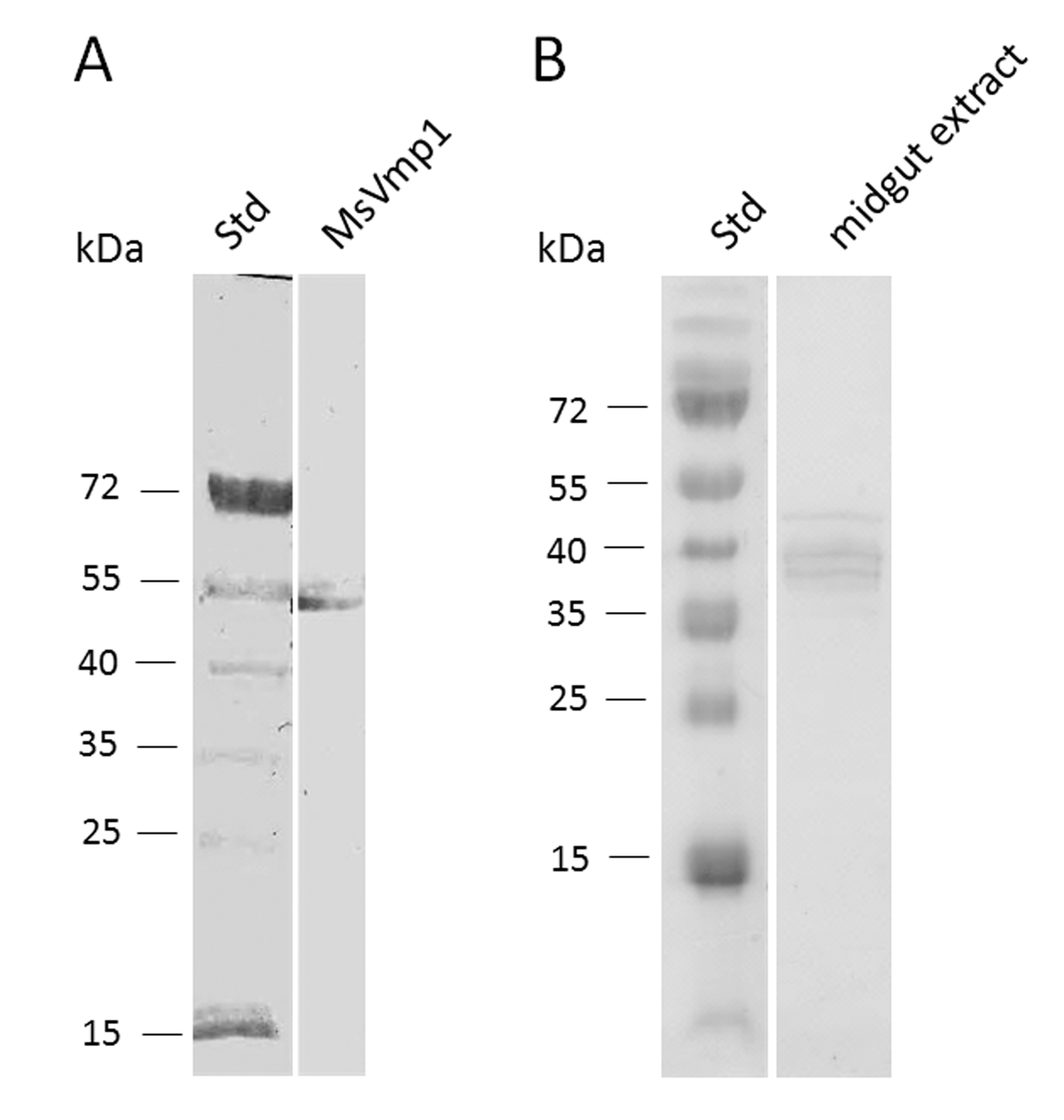

Supplement: Figure S4 — Immunoblots to detect MsVmp1 using anti-Vmp1 antibodies. (A) MsVMP1 was expressed in E. coli BL21 cells, purified by Ni-NTA chromatography and separated by SDS-PAGE. (B) Crude extracts from the posterior midgut of M. sexta fifth instar larvae were subjected to SDS-PAGE. The proteins were blotted onto nitrocellulose and reacted with anti-Vmp1 antibodies. Std, standard proteins with indicated molecular masses in kDa. (TIF) [file pone.0082015.s004.tif]

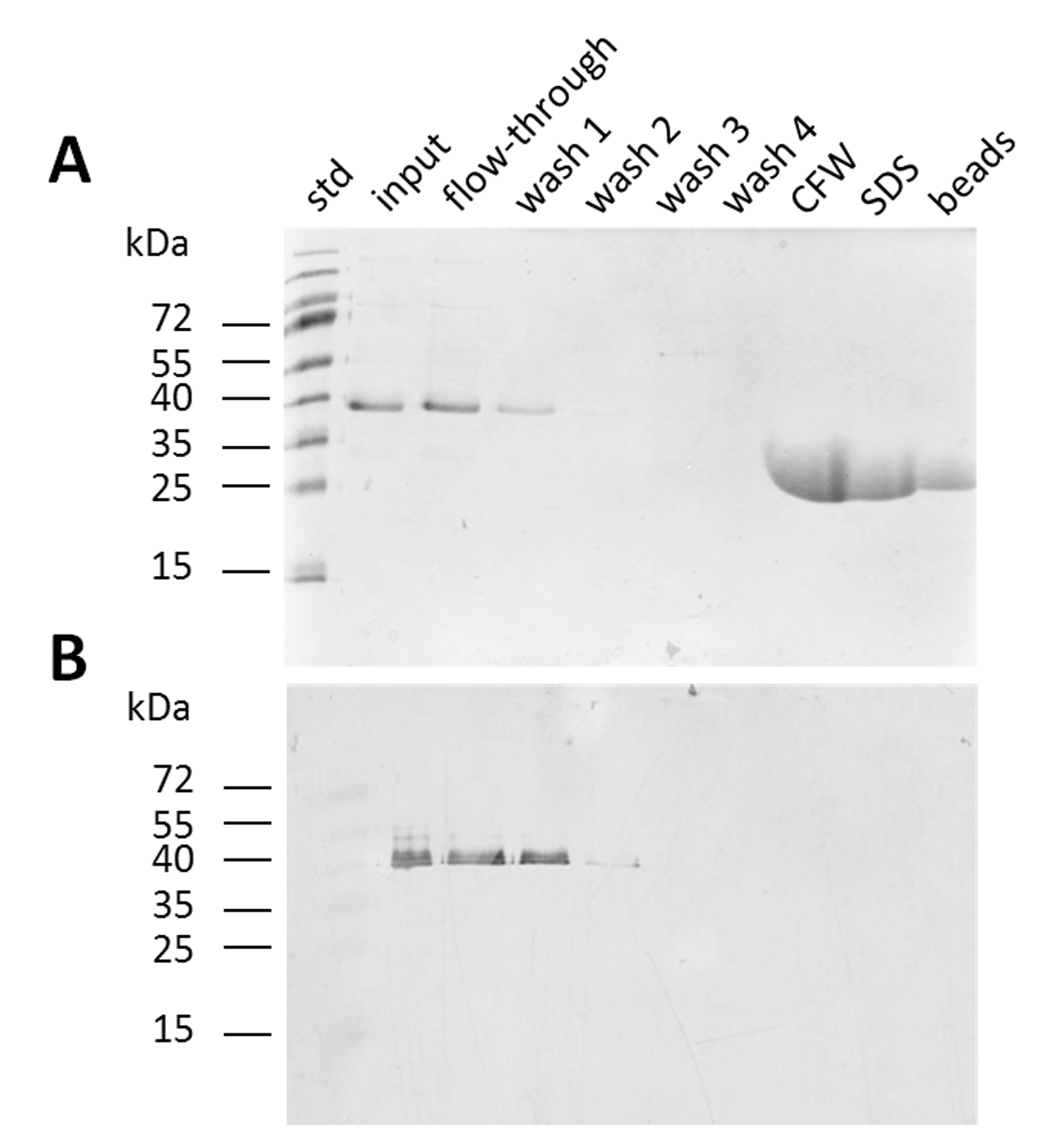

Supplement: Figure S5 — Chitin binding assay reveals no interaction between MsVmp1 and colloidal chitin. Purified, recombinant MsVmp1 (expressed in insect cells) was incubated with chitin beads, transferred to a column, washed and eluted either by SDS or CFW treatment. (A) Aliquots of the resulting flow-through, wash and eluate fractions were separated by SDS-PAGE and stained with Coomassie blue. The smear at around 25 kDa in the eluate fractions is due to the presence of CFW and does not contain proteinaceous material. Specifically, it is not detected by anti-Vmp1 antibodies as shown in the following immunoblot. (B) Aliquots of the fractions were separated by SDS-PAGE, blotted onto nitrocellulose and immune-labeled with anti-VMP1 antibodies. Std, pre-stained standard proteins with indicated molecular masses in kDa. (TIF) [file pone.0082015.s005.tif]

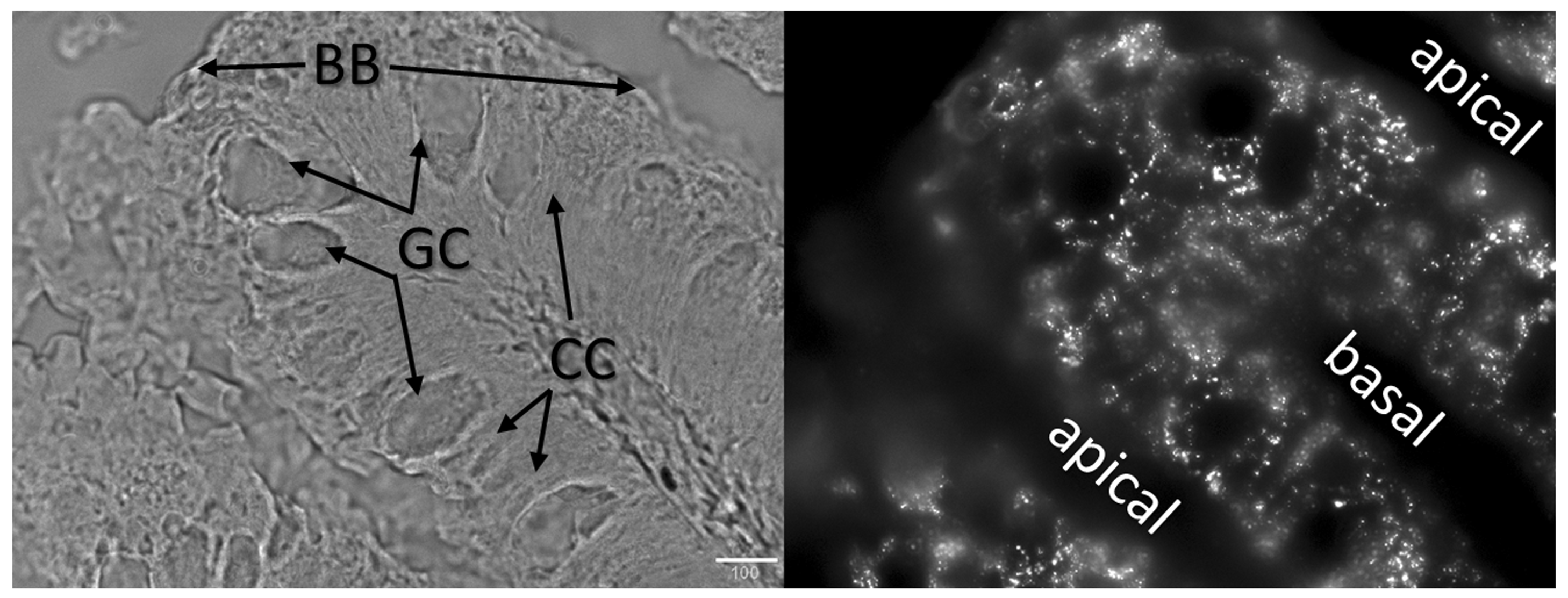

Supplement: Figure S6 — Immune-detection of MsVmp1 in the posterior midgut from 5th instar larvae of feeding M. sexta. Left panel, a 20 µm cryosection of the posterior midgut was imaged by DIC microscopy. Right panel, the cryosection was stained with anti-Vmp1 antibodies. The primary antibodies were detected with ALEXA 488-conjugated anti-guinea pig IgGs. Antibody signals were visualized using an inverse fluorescence microscope and appropriate filters for excitation and emission tag. BB, brush border; GC, goblet cell; CC, columnar cell; Size bar, 100 µm. (TIF) [file pone.0082015.s006.tif]

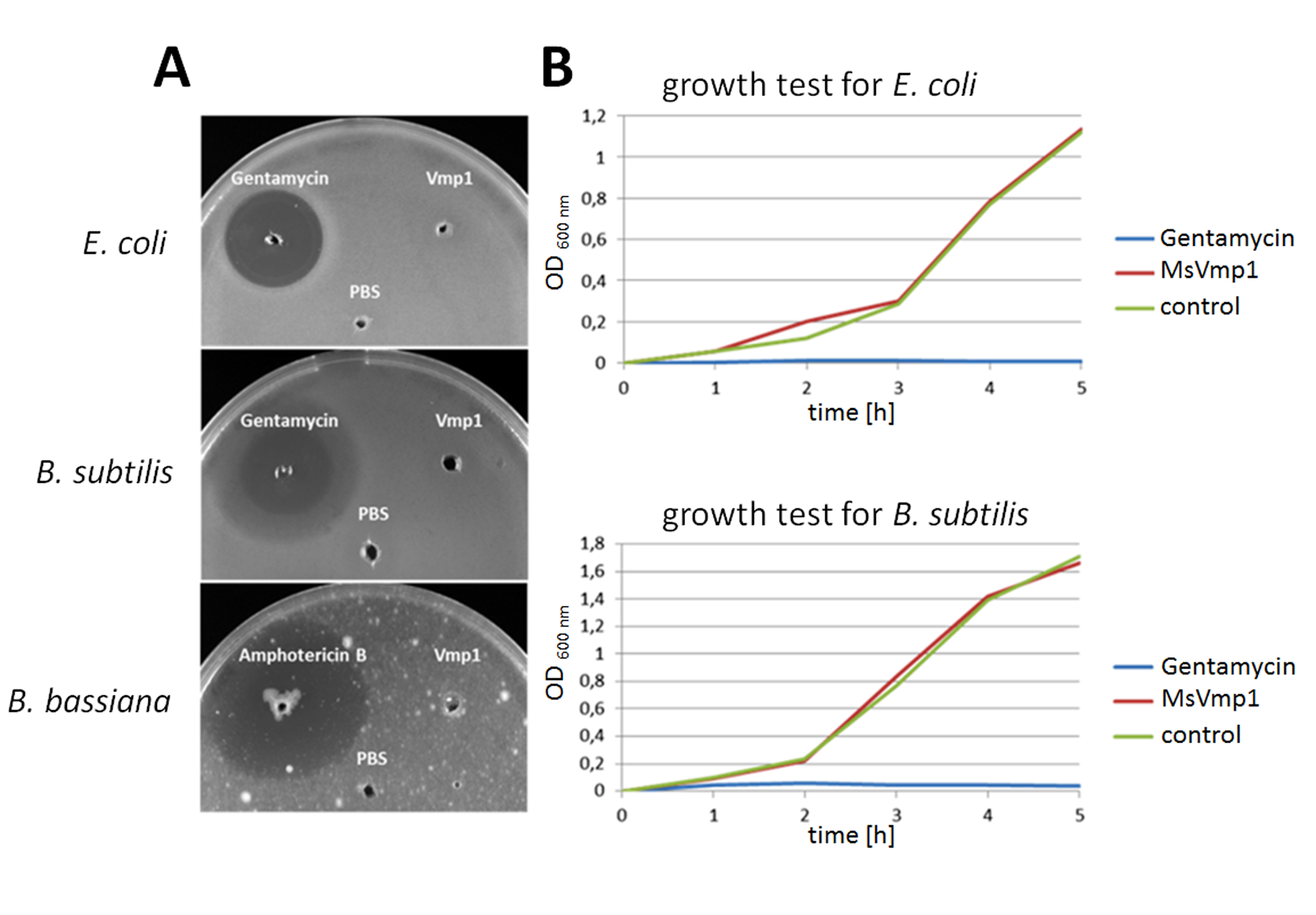

Supplement: Figure S7 — Anti-microbial activity assays. MsVmp1 (expressed in insect cells) was assayed for antimicrobial activity. (A) Disc diffusion assay using 2 µg/ml of MsVmp1 on agar plates inoculated with gram (-) E. coli DH5α, gram (+) B. subtilis,and the fungus B. bassiana at titers yielding confluent growth. As positive controls, the antibiotics Genatmycin and Amphotericin B were used. MsVmp1 did not exhibit antimicrobial activity. (B) Growth tests in the presence and absence (-) of MsVmp1 (2 µg/ml). Gentamycin was used to inhibit the growth of gram (-) E. coli and gram (+) B. subtilis. (TIF) [file pone.0082015.s007.tif]
