# Supplementary material for: A Lepidopteran-Specific Gene Family Encoding Valine-Rich Midgut Proteins
Source: PLoS One. 2013 Nov 29;8(11):e82015. doi: 10.1371/journal.pone.0082015 (PMC3843731; doi:10.1371/journal.pone.0082015)
Supplement: Table S1 — Primer sequences. (DOCX) [file pone.0082015.s008.docx]

Table S1. Primer sequences

| **Primer** | **Sequence (5’ → 3’)** |
| --- | --- |
| **pET29b VMP1-F** | 5’-TACTCAGGATCCGAAATTCCTGTTGATAACC-3’ |
| **pET29b VMP1-R** | 5’-TACTCAGAATTCGCAGAACGGGCAAGGG3’ |
| **pFBD-VMP1-F** | 5’-TACTCAGGATCCTATGAAATTCCTGTTGATAACC-3’ |
| **pFBD-VMP1-R** | 5’-TACTCAAAGCTTCTAATGGTGATGATGGTGATGGTGATGAGAACGGGCAAGG-3’ |
| **M13-F** | 5’-GTTTTCCCAGTCACGAC-3’ |
| **M13-R** | 5’-CAGGAAACAGCTATGAC-3’ |
| **VMP1-F** | 5’-ACCGCTGTACCCACCAAACGCTTC-3’ |
| **VMP1-R** | 5’-AAGAACGGGCAAGGGGGTTTACGGGCTT-3’ |
| **VMP2-F** | 5’-TCATCGTTGCTTTCGCTG-3’ |
| **VMP2-R** | 5’-TCCACAACGTTCACGGGATC-3’ |
| **VMP3-F** | 5’-GTGAACGTTGTGGACATCACC-3’ |
| **VMP3-R** | 5’-GCGACCACAGGGCTGATGTC-3’ |
| **VMP4-F** | 5’-TCTCCCATCTCAGTCGGA-3’ |
| **VMP4-R** | 5’-TTAGTTCAGTTCCTCGGGGAGGGTG -3’ |
| **VMP5-F** | 5’-TTGCCTTCCCCAACAAGCCCGCTGTCAA-3’ |
| **VMP5-R** | 5’-TTGGGGTTTCTCAACTTCCGCAATAGC-3’ |
| **VMP6-F** | 5’-TGCCGGGCATCGGTGCTGCCGGAC-3’ |
| **VMP6-R** | 5’-CTCGGGGACAACAACTTCAGG-3’ |
| **VMP7-F** | 5’-TGATCGTTGCCGTGGCTTC-3’ |
| **VMP7-R** | 5’-CGGCAGCAGGGCCGGGCAT-3’ |
| **VMP8-F** | 5’-GTCATCTTTGACCAGGAGTC-3’ |
| **VMP8-R** | 5’-CAACCTGGTCAACAATGTGCAC-3’ |
| **VMP9-F** | 5’-CATCTCCGCTGATTTCGC-3’ |
| **VMP9-R** | 5’-ACAGCGACGGGCTCAATGA-3’ |
| **Ms-RPS3-F** | 5’-TACGCTGAGAAAGTTGCC-3’ |
| **Ms-RPS3-R** | 5’-CATGGACTTGGCTCTCTG-3’ |
| **T7-pBlueskript-F** | 5’-AATACGACTCACTATAGGGC-3’ |
| **T3-pBlueskript-R** | 5’-ATTAACCCTCACTAAAGGGA-3’ |
