# Supplementary material for: A Lepidopteran-Specific Gene Family Encoding Valine-Rich Midgut Proteins
Source: PLoS One. 2013 Nov 29;8(11):e82015. doi: 10.1371/journal.pone.0082015 (PMC3843731; doi:10.1371/journal.pone.0082015)
Supplement: Table S2 — Blastp search for MsVmp orthologs in different insect orders and other taxonomic groups. The accession numbers of proteins revealing the best hits (lowest E values) within the indicated group are given. (DOCX) [file pone.0082015.s009.docx]

Table S2. Blastp search for *MsVmp* orthologs in different insect orders and other taxonomic groups. The accession numbers of proteins revealing the best hits (lowest E values) within the indicated group are given.

| **Order** | **Species** | **Accesion No.** | **Total score** | **E value** |
| --- | --- | --- | --- | --- |
| **Archaeognatha (taxid:29994)** | *Pedetontus saltator* | ACY43778.1 | 18.9 | 4.8 |
| **Zygentoma (taxid:30264)** | *Thermobia domestica* | CBH30979.1 | 26.2 | 0.005 |
| **Ephemeroptera (taxid: 30073)** | *Ephemerella inconstans* | ACY43854.1 | 19.2 | 28 |
| **Odonata (taxid: 6961)** | *Libellula pulchella* | ADY75708.1 | 19.6 | 14 |
| **Plecoptera (taxid:50622)** | *Oyamia lugubris* | BAJ78706.1 | 18.9 | 23 |
| **Blattodea (taxid:85823)** | *Rhyparobia maderae* | AGA01579.1 | 21.2 | 5.0 |
| **Mantodea (taxid: 7504)** | *Tenodera aridifolia* | BAJ78710.1 | 35.0 | 0.36 |
| **Isoptera (taxid:7499)** | *Coptotermes formosanus* | AGM32834.1 | 21.9 | 3.3 |
| **Grylloblattodea (taxid:58557)** | *Galloisiana yuasai* | BAJ78701.1 | 34.6 | 0.44 |
| **Mantophasmatodea (taxid:192413)** | *Karoophasma biedouwens* | BAJ78702.1 | 48.5 | 1.7 |
| **Dermaptera (taxid:27434)** | *Challia fletcheri* | YP_006665720.1 | 20.0 | 1.5 |
| **Orthoptera (taxid: 6993)** | *Gryllus bimaculatus* | AGJ83078.1 | 20.8 | 24 |
| **Phasmatodea (taxid:7020)** | *Phraortes illepidus* | BAJ78699.1 | 35 | 1.4 |
| **Embiotera (taxid: 50657)** | *Aposthonia japonica* | BAJ78656.1 | 33.1 | 0.44 |
| **Zoraptera (taxid:30265)** | *Zorotypers sp.* | BAJ78665.1 | 57.7 | 0.095 |
| **Psocoptera (taxid: 30259)** | *Metylophorus sp*. | BAJ78719.1 | 18.9 | 0.36 |
| **Phthiraptera (taxid:85819)** | *Pediculus humanus corporis* | XP_002432519.1 | 28.5 | 1.5 |
| **Hemiptera/ Homoptera (taxid: 7524)** | *Riptortus pedestris* | BAN20625.1 | 25.8 | 2.9 |
| **Thysanoptera (taxid:30262)** | *Gynaikothrips ficorum* | BAJ78714.1 | 18.9 | 16 |
| **Megaloptera (taxid:50553)** | *Protothermes grandis* | BAJ78722.1 | 18.9 | 2.7 |
| **Raphidioptera (taxid:50482)** | *Inocellia japonica* | BAJ78723.1 | 33.9 | 1.4 |
| **Neuroptera (taxid: 7516)** | *Chrysoperla nipponensis* | BAJ78724.1 | 18.9 | 19 |
| **Coleoptera (taxid: 7041)** | *Dendroctonus ponderosae* | ENN80948.1 | 26.9 | 2.3 |
| **Strepsiptera (taxid:30261)** | *Stichotrema asahinai* | BAJ78725.1 | 18.5 | 5.9 |
| **Mecoptera (taxid: 27420)** | *Panorpa takenouchii* | BAJ78730.1 | 18.9 | 5.9 |
| **Siphonaptera (taxid: 7509)** | *Xenopsylla cheopis* | ABM55409.1 | 19.6 | 4.3 |
| **Diptera (taxid:7147)** | *Drososphila melanogaster* | NP_726016.1 | 30.0 | 1.3 |
| **Trichoptera (taxid: 30263)** | *Triplectides jaffueli* | CBI70947.1 | 22.3 | 0.011 |
| **Lepidoptera (taxid: 7088)** | *Bombyx mori* | ACY06933.1 | 65.9 | 4e-14 |
| **Hymenoptera (taxid: 7399)** | *Nasonia vitripennis* | XP_001602588.2 | 28.9 | 1.6 |
| **Crustaceae (taxid:6657)** | *Daphnia pulex* | EFX85274.1 | 32.0 | 9e-05 |
| **Fish (taxid:7898)** | *Oreochromis niloticus* | XP_003452823.1 | 27.3 | 10 |
| **Reptilia (taxid:8504)** | *Anolis carolinensis* | XP_003219825.1 | 29.3 | 0.38 |
| **Aves (taxid: 8782)** | *Taeniopygia guttata* | XP_002192978.2 | 33.5 | 0.065 |
| **Mammalia (taxid:40674)** | *Cavia porcellus* | XP_003474605.1 | 32.0 | 1.5 |
